# Improved Predictability of Diagnosis and Prognosis Using Serum‐ and Tissue‐Derived Extracellular Vesicles From Bulk mRNA Sequencing in Pancreatic Ductal Adenocarcinoma

**DOI:** 10.1002/cam4.70538

**Published:** 2025-01-15

**Authors:** Qian Zhu, Zhang Chen, Ming Tian, Xin Yan, Xiangdong Gongye, Zhicheng Liu, Anbang Zhao, Zhiyong Yang, Yufeng Yuan

**Affiliations:** ^1^ Department of Hepatobiliary and Pancreatic Surgery Zhongnan Hospital of Wuhan University Wuhan China; ^2^ Clinical Medicine Research Center for Minimally Invasive Procedure of Hepatobiliary & Pancreatic Diseases of Hubei Province Wuhan China; ^3^ TaiKang Center for Life and Medical Sciences Wuhan University Wuhan China

**Keywords:** diagnosis, Exosomal miRNA, exosome, PDAC, prognosis

## Abstract

**Background:**

Early‐stage pancreatic ductal adenocarcinoma (PDAC) is frequently misdiagnosed, contributing to its high mortality rate. Exosomal microRNAs (miRNAs) have emerged as potential biomarkers for the early detection of PDAC.

**Aims:**

This study aimed to evaluate the feasibility of using exosomal miRNAs from PDAC tissues and serum as biomarkers for early detection and prognosis.

**Materials & Methods:**

Exosomes were isolated from healthy individuals and PDAC patients via tissue and serum samples, then identified by analyzing their particle size and protein content. PDAC‐specific exosomal miRNAs were identified using a microRNA array. A large cohort was subsequently recruited to validate these findings. The diagnostic capacity of the identified miRNAs was assessed using the Brier score and area under the curve (AUC). Verified miRNAs were also used to confirm intracellular mRNA change patterns.

**Results:**

The combination of miR142‐3p, miR148a‐3p, and CA199 showed a higher AUC (0.747) compared to CA199 alone (0.716) in ROC curve analysis. Gene Ontology (GO) annotations revealed that the two‐miRNA panel was associated with multiple oncogenic pathways.

**Discussion:**

142‐3p and miR148a‐3p were identified as specific to PDAC and, when combined with CA199, improved diagnostic accuracy. Their involvement in oncogenic pathways underscores their relevance as diagnostic and prognostic biomarkers.

**Conclusion:**

MiR142‐3p and miR148a‐3p, alongside CA199, show promise as non‐invasive biomarkers for early detection and prognosis of PDAC, improving diagnostic accuracy.

## Graphical abstract

Combined analysis of the content from tissue‐derived EVs and serum‐derived EVs can better identify early biomarkers of pancreatic ductal adenocarcinoma (PDAC). Our study identifies two specific miRNAs, miR142‐3p and miR148a‐3p, as diagnostic serum biomarkers for PDAC. Tissue‐derived EVs are found in the interstitial space and are important for intercellular communication. These results indicate that the combination of miR142‐3p, miR148a‐3p, and CA199 was more accurate than CA199 alone in predicting clinical outcomes. Combined analysis of tissue‐derived EVs with serum‐derived EVs could be more accurate, as tissue‐derived EVs could be more specific than serum‐derived EVs.

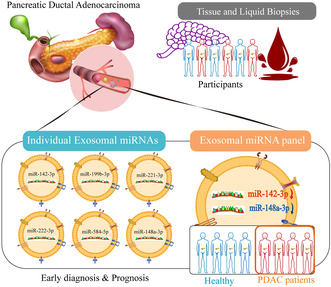

## 1 Introduction

One of the most fatal conditions in the world is pancreatic ductal adenocarcinoma (PDAC) [1]. Recent scientific development has allowed a significant advancement in understanding the molecular processes that are involved in PDAC and its treatment; however, a sensitive biomarker that would aid in early detection or furnish an effective drug target for treatment is still undetermined. The mortality rate of PDAC is very close to 100% [2, 3]. Therefore, there is an urgent need to identify the factors associated with PDAC progression and novel specialized therapeutic targets for PDAC patients [4].

Currently, noninvasive markers that can improve the diagnosis of pancreatic cancer are urgently needed [5]. Various studies focusing on the application of serum‐derived extracellular vesicles (Se‐EVs) for tumor diagnosis have been recently reported [6, 7, 8]. Se‐EVs are derived from various cells, resulting in a complex composition, and whether the identified RNA biomarkers are tumor‐specific has not been elucidated [9]. The tumor microenvironment includes tumor, structural, and immune cells; however, when working in vitro, cell line culture lacks the complexity of the tumor microenvironment and is not fully representative of tumors. Extracellular vesicles (EVs) from tumor tissue contain more information. EVs can be isolated from the blood circulation and are a source of information for multiple organs and cells. Thus, a detailed classification of several EV subsets directly isolated from tumor tissue can provide better insight into their function in tumor development, leading to the detection of biomarkers. Hopefully, in the future, small vesicle subpopulations produced by different cells, including fibroblasts, inflammatory cells, or cancer cells, in tumor tissue could also be explored. For now, EVs can be extracted not only from the blood of PDAC patients but also directly from PDAC tissues and represent a real and specific response of tumors to the local microenvironment.

Tissue‐derived extracellular vesicles (Ti‐EVs) are gaining increasing attention in the scientific world [10]. EVs are more stable in tissues and can protect their contents, such as RNA and proteins, from degradation or denaturation; however, obtaining tissue samples for diagnosing pancreatic cancer often involves invasive procedures such as puncture biopsy or surgical resection, whereas more reliable and convenient diagnostic methods have been developed using the same acquired tissue. Serum‐based EVs offer the advantage of being relatively noninvasive, being rich in markers, and reflecting systemic conditions. Nonetheless, serum‐based diagnostic approaches for pancreatic cancer are not yet well developed, which calls for the development of a dependable diagnostic method utilizing serum‐derived EVs. In this investigation, we isolated Se‐EVs and Ti‐EVs from PDAC patients to identify the most accurate biomarkers and determine a diagnostic strategy with increased EV discriminatory capability by integrating transcriptomic and proteomics molecular serum markers.

## 2 Materials and Methods

### 2.1 Experimental Workflows

#### 2.1.1 Tissue and Blood Sample Collection

**FIGURE 1:**
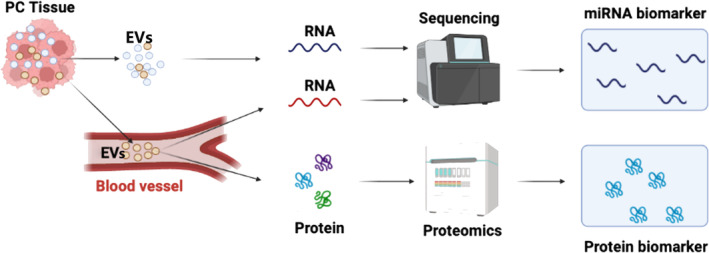
Experimental workflows were employed in this study to evaluate the biomolecular landscape of EVs.

Eight patients who were subjected to surgery in 2020 were recruited from the HBP department of Wuhan University Zhongnan hospital (Table 1; Figure 1). Three healthy donors were also included in this study. All tumor tissue and blood were collected after informing the participants about the study and after written consent was signed by them. All the protocols in this investigation were authorized by the Ethical Committees of Wuhan University Zhongnan hospital. Table 1 shows the participants' medical information.

**TABLE 1:** Clinical characteristics of PDAC patients.

Sample	Age (year)	Gender (M = male, F = female)	Cholecystitis	Jaundice	Emaciation	Reduce jaundice	CA199 (μ/ml)	Tumor size (cm)	TNM stage	Capsule invasion	Fat infiltration	Vascular invasion	Blood transfusion	Postoperative bleeding
T1‐N1	75	M	N	N	N	N	> 1000.0	4 × 3 × 3	T2N0M0	Y	Y	N	Y	N
T2‐N2	60	M	N	N	N	N	1000	3 × 2 × 2	T2N0M1	Y	Y	Y	Y	N
T3‐N3	60	M	N	N	N	N	40.05	4 × 4 × 3	T2N2M0	Y	Y	Y	N	Y
T4‐N4	58	M	N	N	N	N	1081	4 × 4 × 4	T2N0M0	Y	N	Y	N	N
T5‐N5	69	M	Y	Y	Y	PTCD	> 1000.0	3 × 2 × 1	T2N2M0	N	Y	Y	Y	N
T6‐N6	63	F	N	N	N	N	> 1000.1	3 × 2 × 1	T2N1M0	N	N	Y	N	N
T7‐N7	68	M	N	Y	Y	PTCD	920.10	3 × 2 × 1	T2N0M0	N	N	Y	N	Y
T8‐N8	49	F	Y	Y	N	PTCD	30.53	2 × 2 × 1	T1cN0M0	N	N	N	N	N

Abbreviations: N=no; PTCD=Percutaneous Transhepatic Cholangial Drainage; Y=yes.

### 2.2 EV Isolation From Tissue and Plasma Samples

EVs were isolated from the tissues by reproducing the method previously applied by Vella et al. [11], with minor modifications. A kit (cat. no. 130‐095‐929, Miltenyi Biotec) was utilized for tissue dissociation. R, A, and H enzymes were re‐dissolved according to the protocol given by the manufacturer. The dissociation solution was prepared freshly by mixing RPMI (2.2 mL) with enzymes R (50 μL), H (100 μL), and A (12.5 μL). After being weighed and segmented on dry ice, a 200 mg tissue sample was placed in a dissociation solution (10–15 min, 37°C). The material was filtrered twice (70‐μm filter) for removal of any remaining cells. At 4°C, the filtered and homogenized tissue was centrifuged for 10 min at 300 *g* and the supernatant was re‐centrifuged (2000 ×*g*, 10 min, 4°C) after which the supernatant was filtered (0.22‐μm filter) and re‐centrifuged (10,000 *g*, 20 min, 4°C) followed by ultracentrifugation (UC) for 2 h at 150000 *g* and 4°C. The precipitate was dissolved in 1 mL of PBS, passed through an Exosupur column (Echobiotech, China), and concentrated in an Amicon Ultra spin filter (100 kDa cutoff; Merck, Germany). Size exclusion chromatography (SEC) was carried out to extract EVs from the serum samples followed by ultracentrifugation/ultrafiltration (UC/UF). The UC protocol was performed after optimizing the method previously carried out by Théry C. et al. [12]. To get rid of cellular debris, the 37°C thawed plasma was centrifuged (3000 ×*g*, 15 min). After seven‐fold dilution of the supernatant with PBS, the material was centrifuged (13,000 ×*g*, 30 min), filtered (0.22 μm filter), and ultracentrifuged (100,000 ×*g*, 2 h, 4°C) using a P50A72‐986 rotor (CP100NX; Hitachi, Japan). PBS was used for resuspension of the pellet before 2 h (4°C; 100,000 ×*g*). PBS was used to carefully wash the exosome in the obtained pellet and then re‐dissolved in 100 μL PBS. The EVs from the blood were also separated by the SEC method given by Boing et al. [13] with minor optimizations. In brief, 1 mL of plasma was filtered (0.8 μm), diluted 1.5 times in PBS, and passed through Exosupur columns using 0.01 M PBS for elution, after which the eluted fractions were concentrated as above.

### 2.3 Nanoparticle Tracking Analysis (NTA)

The number and sizes of the particles in 1 × 10^7^/mL and 1 × 10^9^/mL EV suspensions were assessed using a ZetaView PMX 110 (Particle Metrix, Meerbusch, Germany) with a 405 nm laser. A 60‐s 30‐frame‐per‐second video was obtained. The particle velocity was calculated using NTA software (ZetaView 8.02.28). One cubic centimeter of diluted EV samples was introduced into the viewing chamber using a syringe.

### 2.4 Transmission Electron Microscopy (TEM)

Following three TBS washes and 1% uranyl acetate/0.05 Tylose staining, for 2 min, the generated EV (10 μL) was attached to glow‐discharged, ultra‐thin carbon‐coated grids with a 400 mesh screen (EMS CF400‐CU‐UL). After collection and drying, grids were analyzed on an 80 kV Philips CM120 apparatus, and photographs were taken with a high‐resolution 8‐Megapixel AMT XR80 camera (16‐bit). On a copper mesh of 10 μL, the exosome solution was kept for 60 s at room temperature (RT). The exosomes were rinsed with sterile distilled water and stained with uranyl acetate for 1 min. Under incandescent light, the samples were dehydrated for 2 min. Examining of the copper mesh was performed using images taken with a TEM (H‐7650, Hitachi Ltd., Tokyo, Japan).

### 2.5 Western Blotting (WB)

Fractions containing EVs were lysed with RIPA buffer, with subsequent quantification of proteins with a BCA kit (Thermo Fisher). On 4 to 15% pre‐cast gradient SDS‐PAGE non‐reducing gels from Bio‐Rad, the same quantity of total EVs and BH proteins were separated, and they were then transferred to PVDF membranes (Sigma Aldrich). After that, these membranes were stored at the ambient temperature for 1 h in 5% non‐fat milk (170‐6404, Bio‐Rad). The membranes were stored at 4°C for 24 h with antibodies against CD81 (1:1000), Bip (1:1000), CD63 (1:1000) (BD Biosciences; 610,978 and 556,019, respectively), Rab27 (1:1000) (Santa Cruz Biotechnology; sc74586, sc23962), TSG101 (1:500), Syntenin (1:500), and Calnexin (1:2000 dilution) (Abcam, ab92726, ab125011, ab133267, ab76154, and ab22595, respectively). Following the attachment of the primary antibody, an HRP‐conjugated secondary antibody (1:10000 dilution) was used to incubate the membranes for 1 h at ambient temperature (sc‐2357, sc‐516,102; Santa Cruz Biotechnology). The membranes were rinsed again after 1 h with PBST, and the enzyme‐linked antibodies were observed via Pico chemiluminescent substrate (34,580; Thermo Fisher) and were recorded on a film (Millipore Sigma GE28‐9068‐38).

TSG101 (sc‐13,611, Santa Cruz), Alix (sc‐53,540, Santa Cruz), HSP90 (60318‐I‐Ig, Proteintech), calnexin (10427‐2‐AP, Promega), rabbit polyclonal anti‐CD63 (sc‐5275, Santa Cruz), and CD9 (60232‐I‐Ig, Proteintech) was used to denature the EV supernatant in 5× SDS buffer before WB (10% gel; 50 μg protein per sample).

### 2.6 Quantitative PCR (qPCR)

The total RNA from the Ti‐EVs and Se‐EVs was separated using TRIzol (Invitrogen), and the RevertAid First Strand cDNA Synthesis Kit (Thermo Fisher) was employed to reverse‐transcribe the resulting first‐strand cDNA using a random primer. The cDNA was subsequently examined using the Applied Biosystems QuantStudio 3 Real‐Time PCR equipment. Expression of miRNAs was measured. As an internal control, GAPDH was maintained.

### 2.7 Small RNA Sequencing and Sequencing Data Analysis

The total RNA of Ti‐EVs and Se‐EVs was isolated and purified via Ion Total RNA‐Seq Kit V2 (Life Technologies 4,475,936) by observing the kit's descriptions. Purity and concentrations were assessed using an Agilent Bioanalyzer 2100 System's RNA Nano 6000 Assay Kit (Agilent Technologies, CA, USA). The SMARTer Stranded Total RNA‐Seq Kit V2 (Takara, Japan) input material for sequencing libraries was determined to be RNA concentrations of 250 pg–10 ng from each sample. The index codes provided by the manufacturer and their instructions were included to characterize sequences for every sample. Libraries were constructed using 1–500 ng RNA with a QIAseq miRNA Library Kit (Qiagen, Germany). Primers with unique molecular indices (UMIs) were developed. The library quality was assessed as above. A TruSeq PE Cluster Kitv3‐cBot‐HS (Illumina) was utilized for clustering the index‐coded samples using the acBot Cluster Generation System. Following their clustering, the libraries were sequenced using an Illumina Hiseq system, yielding paired‐end reads.

### 2.8 Mass Spectrometry and Proteomics Data Analysis

The Se‐EVs were lysed using lysis buffer (100 mM NH4HCO3, 0.2% SDS, and 6 M urea), followed by ultrasound treatment on centrifugation (12,000 g, 15 min, 4°C). the supernatant was treated with DTT (10 mM) at 56°C for an hour, all samples with iodoacetamide were alkylated for an additional hour at ambient temperature in the dark and then treated with 4× acetone for 2 h at −20°C. After centrifuging the mixture, removing the pellet, and washing it with cold acetone, it was dissolved in 0.1 M TEAB and 6 M urea. Protein levels were determined using a BCA kit (Product ID 23225, Thermo Fisher), with BSA as standard. Following the concentration of 20 μL of the proteins collected, they were separated at 80 V and 120 V for 20 and 90 min, respectively, on 12% SDS‐PAGE. The protein samples were kept at 37°C overnight in 500 μL of 100 mM TEAB buffer and trypsin (3 μL). A corresponding volume of formic acid (1%) was added to the digested proteins, and they were centrifuged (12,000 *g*) at RT for 5 min. The obtained upper layer was then gradually injected in the C18 desalination column, 3× rinsed with 1 mL of wash reagent (4% acetonitrile, 0.1% formic acid) before eluting two times via elution buffer (0.4 mL; 75% acetonitrile, 0.1% formic acid). After mixing, the eluent was cryopreserved. Before being loaded onto a self‐made C18 (2 cm × 75 μm, 3 μm) nano trap column, solution A was made by mixing the protein powder which was freeze‐dried in 10 μL of 0.1% formic acid in water. It was used to isolate and analyze the peptide. A 0.1% formic acid and 80% acetonitrile (solution B) made up the mobile phase. The sample was eluted at a 600 NL/min flow rate. The content of solution B in the eluent increased from 6% to 100% within 60 min. By incrementing the level of concentrated solvent from 6 B to 100% for more than 60 min. The peptides were evaluated using a Thermo Fisher Q exactive hf‐x mass spectrometer. With a spray voltage of 2.3 kV, the nanospray flextm (ESI) served as the ion source. Utilize the UniProt database (http://www.uniprot.org) to get the original data discovered by MS. Methionine oxidation and N‐terminal acetylation are considered reversible alterations, whereas urea methylation is a stable modifier. The obtained protein contained a minimum of one unique peptide and FDR ≤ 0.01. The identified proteins were evaluated by GO, COG, and KEGG pathway analyses using DAVID. Annotations of the identified protein's biological constituents were improved by Funrich and STRING. By applying Reactome and the KEGG analyses, the mechanisms in which the identified proteins were implicated were elucidated. Using the previously indicated tools, the enrichment's statistical significance was elucidated. For evaluation, only considerable groups (FDR‐corrected *p* < 0.01) were used.

### 2.9 Statistical Analysis

The software SPSS 16.0.0 or R (www.r‐project.org) were used for all statistical analyses. The means ± standard error (SE) or standard deviation (SD) of at least three separate experiments conducted in triplicate are displayed. In bar graphs and scatter plots, error bars indicate SE or SD. The One‐Sample Kolmogorov–Smirnor test was employed to determine if the results were normally distributed. When comparing measurement data between two groups, the paired‐sample *t*‐test or independent‐sample *t*‐test was employed if the data were normally distributed and the variance across the groups was comparable. If the results showed a significant difference, the Student Newman Keuls test was performed to look at the variances between the studied groups. Nonparametric tests were used for comparisons when the data displayed a skewed distribution. The Chi‐squared or Fisher Exact tests were used to analyze the enumerated data. Overall survival was evaluated using Kaplan–Meier curves and Log‐rank tests. According to the selected variables using a univariate analysis, the independent factors were identified using the Cox proportional hazards model. To generate the ROC curves, PDAC patients were classified as either surviving longer or shorter than the median OS; patients who survived for shorter periods than the median OS at the last follow‐up were excluded. Cox regression models were respectively built with no predictive variables, with CA19‐9 only, with miRNA142‐3p/miRNA 148a‐3p only, and with both CA19‐9 and miRNA142/miRNA 148a. To determine the average estimations, a 10‐fold cross‐validation was performed 20×. The integrated Brier score was used to describe the prediction error curves. Two‐tailed *p* < 0.05 were considered significant.

## 3 Results

### 3.1 Detection of Ti‐EVs and se‐EVs in PDAC Patients

**FIGURE 2:**
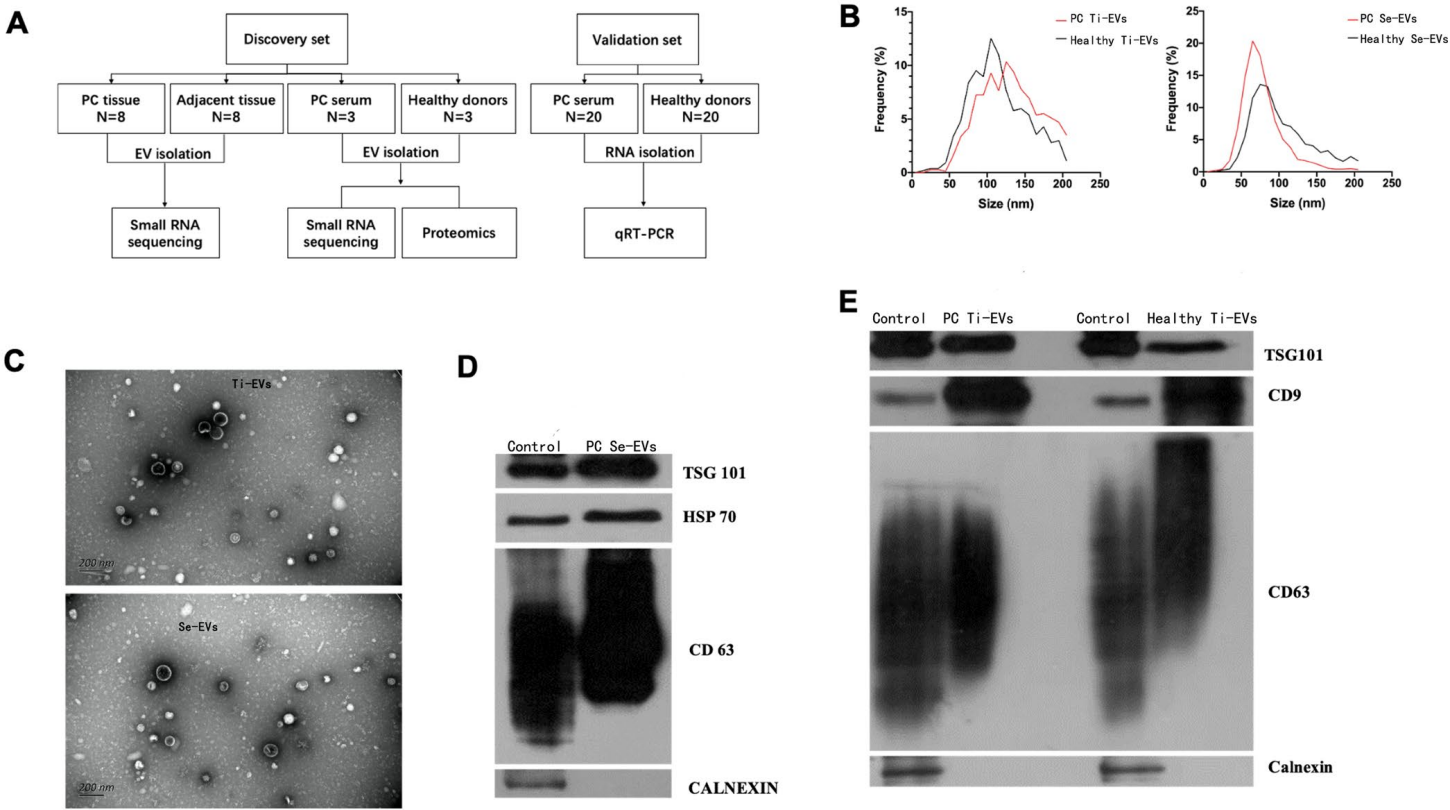
Characterization of Ti‐EVs and Se‐EVs. (A) Investigation design for analyzing PDAC samples from patients and healthy donors. (B) Nanoparticle tracking analysis (NTA) was used to examine the concentration and particle size distribution of the separated Ti‐EVs and Se‐EVs. (C) Transmission electron microscopy of Ti‐EVs and Se‐EVs (Scale bar = 200 μm). (D, E) Western blotting analysis of Ti‐EVs and Se‐EVs proteins to verify the expression of their biomarkers.

The study protocol is presented in Figure 2A. Eight paired Ti‐EV and three paired Se‐EV samples were isolated from PDAC patients. NTA, TEM, and WB analyses were performed with these samples. NTA indicated that the size of Ti‐EVs was larger than that of Se‐EVs (Figure 2B). Both Ti‐EVs and Se‐EVs had a round and cup‐like concave morphology (Figure 2C). TSG101, HSP70, and CD63 were highly expressed in Se‐EVs (Figure 2D), while Ti‐EVs were enriched with TSG101, CD9, and CD63 (Figure 2E). Detailed results are shown in Figure 2. The Supporting Information contain the full‐length original blots (Figure S1).

### 3.2 Differential Expression of miRNAs in EVs

**FIGURE 3:**
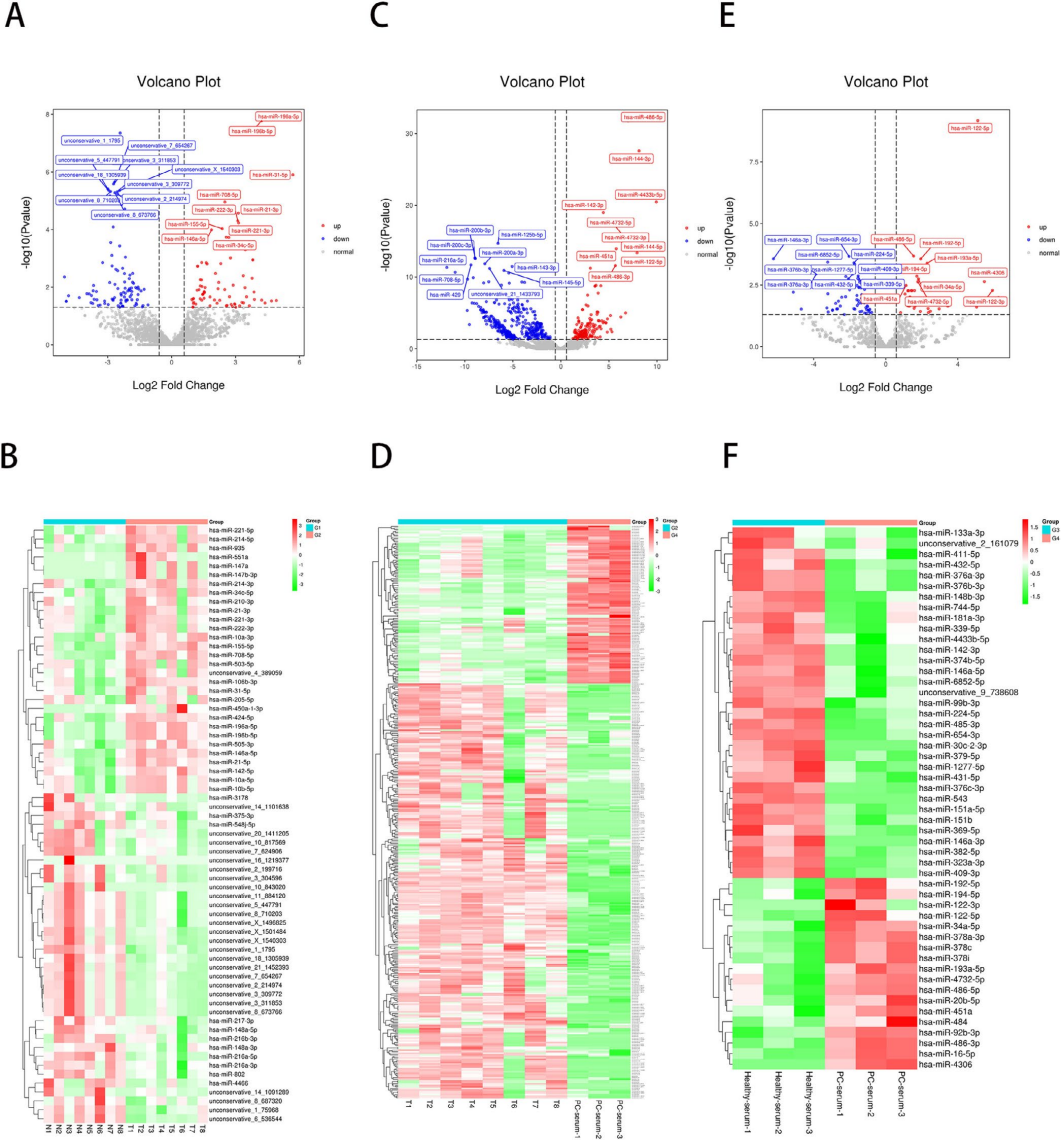
Differential expression of miRNAs in Ti‐EVs and Se‐EVs. (A, C, E) Volcano plots illustrating the differential expression of miRNAs between G1 (Ti‐EVs from para‐cancer tissues) and G2 (Ti‐EVs from PDAC tissues), G3 (Se‐EVs from healthy serum) and G4 (Se‐EVs from PDAC serum), and G2 (PDAC Ti‐EVs) and G4 (PDAC Se‐EVs), respectively. Red dots indicate significantly differentially expressed miRNAs (baseMean ≥ 20 and adjusted p‐value < 0.05), while gray dots represent non‐significant miRNAs. (B, D, F) Heatmaps showing hierarchical clustering of significantly differentially expressed miRNAs for each comparison: G1 vs. G2 (B), G3 vs. G4 (D), and G2 vs. G4 (F). The color gradient represents expression levels, with red indicating upregulation and green indicating downregulation. Clustering patterns reveal distinct miRNA expression profiles between Ti‐EVs and Se‐EVs.

The sequencing of small RNA from 22 samples was performed, and 971.45 M (average per sample = 44.16 M raw reads) were acquired. The total miRNAs detected were 3191, including 1841 known and 1350 newly predicted miRNAs. Bowtie tools software was utilized for clean reads in conjunction with the Silva, Rfam, and GtRNAdb databases, as well as sequence alignment using the Repbase database. Then, filter tRNAs, rRNAs, small nucleolar RNAs (snoRNAs), small nuclear RNAs (snRNAs), and other ncRNA and repeats. Known miRNAs were identified with miRbase and others were determined against the Human Genome (GRCh38). After converting the determined UMI counts of miRNAs to counts per million (CPM), the relative log expression was computed using the EdgeR module. Table S1 and Figure S2 present unannotated reads and the proportion of biotype RNA distribution for each sample. The distribution map reflects the overall expression pattern of miRNAs in the four groups (Figure S3). G1 represents eight Ti‐EVs derived from para‐cancer tissues, G2 represents eight Ti‐EVs derived from PDAC cancer tissues, G3 represents three Se‐EVs derived from healthy serum, and G4 represents three Se‐EVs derived from PDAC patient serum. Differential expression analysis also established that there was only a negligible amount of differentially expressed small RNAs among the four groups (Figure 3). A volcano plot of pairwise comparisons between each group is shown in Figure 3A,C,E. The R 3.5.1 pheatmap package was used to generate heatmaps. Important differentially expressed miRNAs between Ti‐EVs and Se‐EVs were analyzed using heatmap clustering. The candidates for determined miRNAs per pairwise comparison were found using test statistics and *p*‐values (with uncorrected raw *p* < 0.05) (Figure 3B,D,F). The 20 most frequent miRNAs identified in G1 to G4 are shown in Table 2.

**TABLE 2:** Top 20 most abundant miRNAs in G1, G2, G3 and G4.

	Paracancerous Ti‐EV (*n* = 8)				PDAC Ti‐EV (*n* = 8)				Healthy‐Se‐EV (*n* = 3)				PDAC Se‐EV (*n* = 3)		
Rank	miRNAs	Average	SD	Rank	miRNAs	Average	SD	Rank	miRNAs	Average	SD	Rank	miRNAs	Average	SD
1	hsa‐let‐7c‐5p	196544.9	47,729	1	hsa‐let‐7c‐5p	150226.7	26238.83	1	hsa‐miR‐16‐5p	138691.6	13567.78	1	hsa‐miR‐16‐5p	267237.6	33374.18
2	hsa‐let‐7b‐5p	145692.8	45482.61	2	hsa‐let‐7b‐5p	105097.2	21478.05	2	hsa‐let‐7a‐5p	117903.7	8236.54	2	hsa‐let‐7c‐5p	90775.5	11075.78
3	hsa‐let‐7a‐5p	84043.99	15207.23	3	hsa‐let‐7a‐5p	86710.16	22256.21	3	hsa‐let‐7f‐5p	115620.5	8101.98	3	hsa‐let‐7a‐5p	86354.04	13064.91
4	hsa‐let‐7f‐5p	74000.01	15195.25	4	hsa‐let‐7f‐5p	71564.2	12168.73	4	hsa‐let‐7c‐5p	86191.64	5977.15	4	hsa‐let‐7f‐5p	84075.55	12757.24
5	hsa‐let‐7e‐5p	53927.83	12342.39	5	hsa‐miR‐125b‐5p	69904.35	15219.23	5	hsa‐miR‐126‐3p	78814.69	13425.59	5	hsa‐miR‐126‐3p	53046.66	7738.05
6	hsa‐miR‐148a‐3p	51370.44	62857.66	6	hsa‐miR‐125a‐5p	54451.89	23295.78	6	hsa‐let‐7e‐5p	57946.37	3047.46	6	hsa‐let‐7e‐5p	47961.86	6469.42
7	hsa‐miR‐375‐3p	33738.11	14954.49	7	hsa‐miR‐16‐5p	49636.83	26532.73	7	hsa‐miR‐142‐3p	32026.98	1898.23	7	hsa‐miR‐122‐5p	43642.74	31931.99
8	hsa‐miR‐125b‐5p	29612.99	13748.53	8	hsa‐let‐7e‐5p	48582.39	8904.47	8	hsa‐let‐7b‐5p	27785.34	3326.59	8	hsa‐let‐7b‐5p	43155.4	8105.64
9	hsa‐miR‐125a‐5p	29014.21	12948.95	9	hsa‐miR‐21‐5p	39246.81	15067.85	9	hsa‐miR‐223‐3p	23149.26	2724.58	9	hsa‐miR‐486‐5p	35957.14	4808.38
10	hsa‐miR‐16‐5p	28095.01	17544.58	10	hsa‐miR‐199b‐3p	28658.48	8806.64	10	hsa‐let‐7i‐5p	21722.42	3576.56	10	hsa‐let‐7i‐5p	18809.62	1954
11	hsa‐miR‐21‐5p	17404.96	10658.71	11	hsa‐let‐7i‐5p	23790.44	7104.74	11	hsa‐miR‐26a‐5p	17903.26	2569.98	11	hsa‐miR‐92a‐3p	17242.45	4731.97
12	hsa‐miR‐30a‐5p	14783.58	11169.1	12	hsa‐miR‐148a‐3p	17719.25	9633.78	12	hsa‐miR‐26b‐5p	16564.77	440.75	12	hsa‐miR‐142‐3p	13746.26	4382.39
13	hsa‐miR‐30d‐5p	13290.85	10339.7	13	hsa‐miR‐30a‐5p	12913.58	5616.17	13	hsa‐miR‐30a‐5p	16276.34	2894.76	13	hsa‐miR‐223‐3p	12149.8	5996.89
14	hsa‐miR‐199b‐3p	12987.73	7696.86	14	hsa‐miR‐143‐3p	12237.98	2740.75	14	hsa‐miR‐486‐5p	14131.89	7478.02	14	hsa‐miR‐26b‐5p	10546.83	1099.47
15	hsa‐miR‐200c‐3p	11987.82	4571.35	15	hsa‐miR‐26a‐5p	12107.76	4632.87	15	hsa‐miR‐21‐5p	14088.43	1157.43	15	hsa‐miR‐30a‐5p	9665.49	3170.22
16	hsa‐let‐7i‐5p	11754.38	7221.2	16	hsa‐miR‐30d‐5p	11767.32	5290.16	16	hsa‐miR‐92a‐3p	12837.61	490.04	16	hsa‐miR‐26a‐5p	9212.99	1275.26
17	hsa‐miR‐26a‐5p	10861.61	5075.68	17	hsa‐miR‐200c‐3p	10423.16	5727.67	17	hsa‐miR‐103a‐3p	11525.88	1076.32	17	hsa‐miR‐21‐5p	8424.82	1807.66
18	unconservative_21_1433793	9375.47	12413.94	18	hsa‐miR‐7‐5p	9068.17	5180.85	18	hsa‐miR‐107	11525.88	1076.32	18	hsa‐miR‐103a‐3p	7184.74	3476.05
19	hsa‐miR‐7‐5p	8676.53	8574.8	19	hsa‐miR‐26b‐5p	8632.75	2230.45	19	hsa‐miR‐30d‐5p	10532.92	2324.49	19	hsa‐miR‐107	7184.74	3476.05
20	hsa‐miR‐26b‐5p	7288.06	1699.31	20	hsa‐miR‐375‐3p	8520.64	6502.66	20	hsa‐miR‐146a‐5p	10316.04	2206.58	20	hsa‐miR‐93‐5p	6162.78	1887.03

### 3.3 KEGG And GO Analyses

**FIGURE 4:**
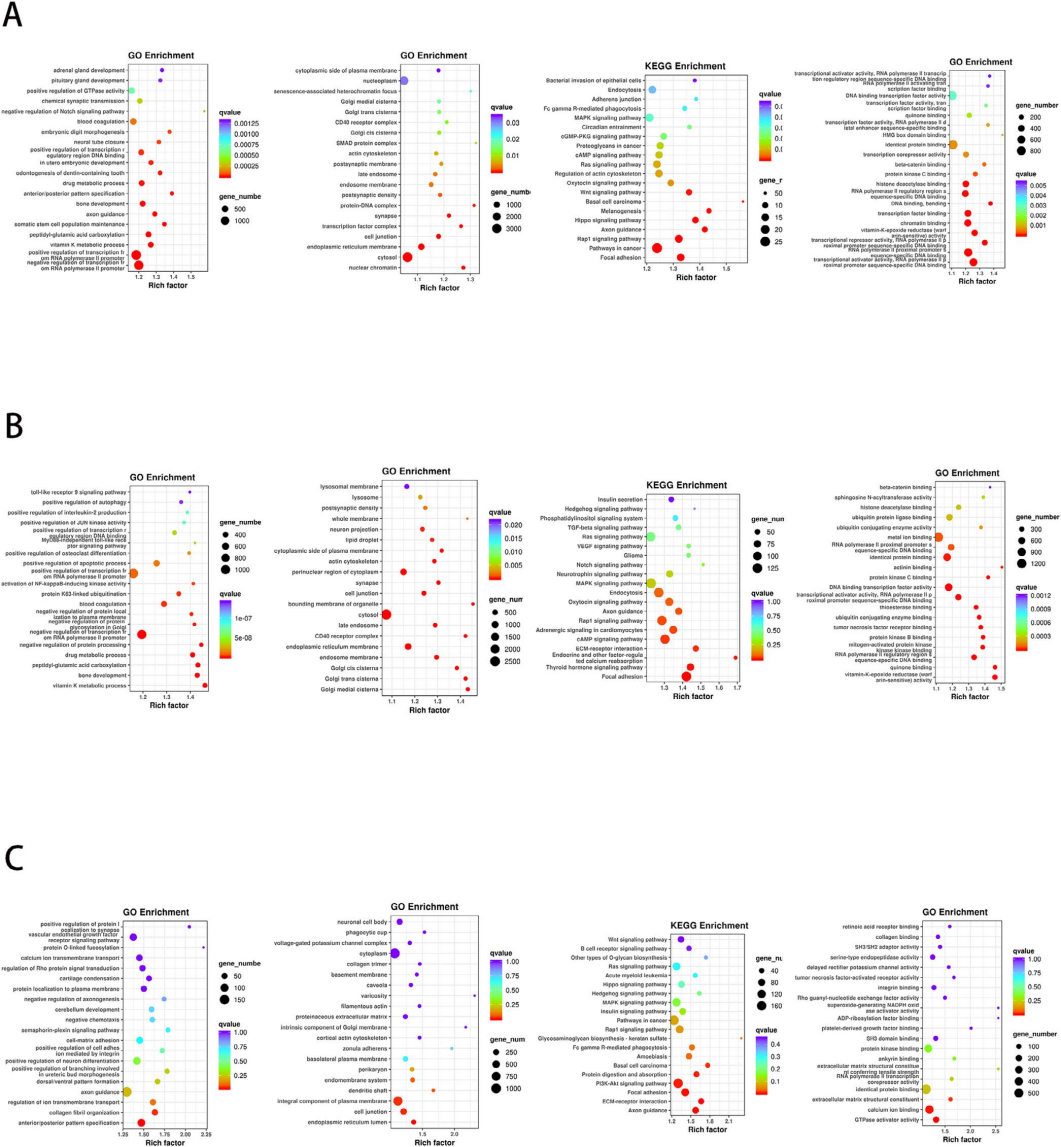
Functional enrichment analysis of miRNA target genes in Ti‐EVs and Se‐EVs. From left to right, the results are shown for molecular function (MF), cellular component (CC), KEGG pathway enrichment, and biological process (BP). (A) G1 vs. G2. (B) G1 vs. G4. (C) G2 vs. G4.

Gene annotations were conducted using BLAST against the NR and SwissProt databases, as well as KEGG, COG, GO, KOG, and Pfam analyses. Of the 19,961 target genes, we were able to acquire annotation information for 19,920 genes. The annotated miRNA target genes for different samples are depicted in Table 3 with differential miRNA target genes shown in Table S2. GO enrichment and KEGG enrichment of Ti‐EVs and Se‐EVs are shown in Figure 4.

**TABLE 3:** Target gene annotation results.

Anno_Database	Annotated_Number	300 ≤ length < 1000	Length ≥ 1000
COG_Annotation	6231	259	5972
GO_Annotation	18,523	1283	17,213
KEGG_Annotation	19,165	1376	17,764
KOG_Annotation	13,245	685	12,556
Pfam_Annotation	18,562	1406	17,129
Swiss‐Prot_Annotation	19,755	1531	18,192
eggNOG_Annotation	19,379	1383	17,975
NR_Annotation	19,851	1581	18,236
All_Annotated	19,920	1626	18,258

### 3.4 Proteome Landscape of EVs Derived From Tissues and Serum Samples

**FIGURE 5:**
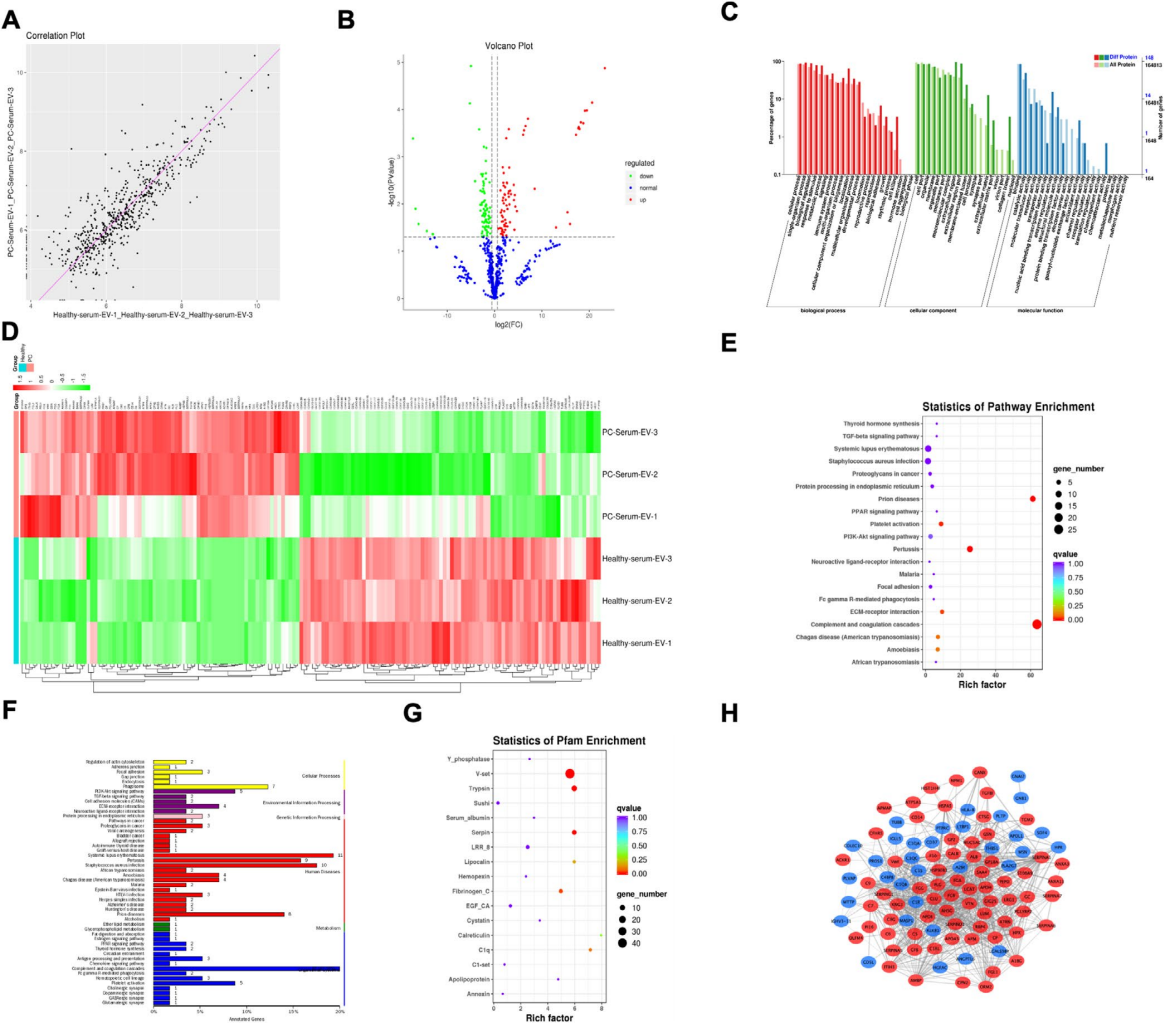
Proteome landscape of EVs derived from tissues and serum samples. (A) Correlation plot of PDAC Se‐EV and healthy donor's Se‐EV. (B) Volcano plot of differentially expressed proteins in PDAC Se‐EV and healthy donor's Se‐EV. (C) Statistical results of GO classification of variably expressed proteins. (D) Heatmap clustering the variably expressed proteins. (E) Results of pathway enrichment statistics. (F) The annotation results of KEGG of differentially expressed proteins. (G) Statistics of Pfam enrichment of differentially expressed proteins. (H) PPI results of differentially expressed proteins.

The serum samples were initially subjected to EV purification and enrichment. Then, EV‐related proteins were extracted, quantified, and subjected to SDS‐PAGE electrophoresis and assay, quality control before mass spectrometry, qualified, enzymatically digested, and then analyzed on a mass spectrometer. The resulting raw files were detected based on mass spectrometry, and a database search was performed, followed by protein identification and subsequent bioinformatics analysis. log2 (FC) ≥ 0.5849625 and P‐level ≤ 0.05 were applied as the criteria for screening during protein expression analysis. Approximately 158 detected proteins exhibited comparable expression levels, of which 76 proteins were up‐regulated and 82 were down‐regulated. The correlation plot of PDAC Se‐EVs and healthy donor Se‐EVs is shown in Figure 5A. The expression level differences in differentially expressed proteins of the two groups samples were visualized by a volcano plot (Figure 5B). Statistical data of the GO classification of differentially expressed proteins between samples are shown in Figure 5C. Hierarchical clustering assessment was carried out on the variably expressed filtered and same/similar expressed proteins (Figure 5D). Statistics of the pathway enrichment results are shown in Figure 5E. The KEGG analysis annotation results for variably expressed proteins were classified based on KEGG pathway type (Figure 5F). The Pfam database is a system that most comprehensively classifies protein domain annotations. Proteins are composed of one or more domains, and the protein sequence of each specific domain has some conservation. Pfam classifies the domains of proteins into different protein families, and the HMM statistical models for the sequence of amino acids of each family align the protein sequences. Statistics of Pfam enrichment of differentially expressed proteins were analyzed (Figure 5G). The differentially expressed protein binding network was developed by contrasting the results of the differential expression protein analysis with STRING database interactions (Figure 5H result missing). Among the differentially expressed proteins, the top six up‐regulated proteins in PDAC patients were SERPINA7, VASN, RBP4, KRT74, NPM1, and ACKR1, and detailed variably expressed proteins are shown in the Data S1.

### 3.5 Combined Analysis of Transcriptomics and Proteomics

**FIGURE 6:**
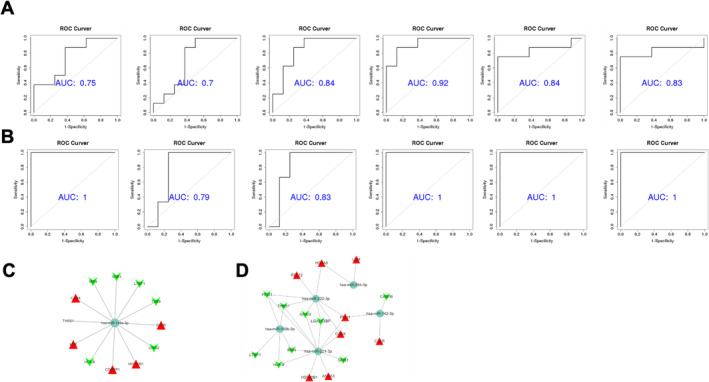
Validation of EV‐derived miRNAs as PDAC biomarkers. (A, B) ROC curves for EV‐derived miRNAs (hsa‐miR‐142‐3p, hsa‐miR‐199b‐3p, hsa‐miR‐221‐3p, hsa‐miR‐222‐3p, hsa‐miR‐584‐5p, and hsa‐miR‐148a‐3p) showing their diagnostic performance as PDAC biomarkers, based on miRNA sequencing of 22 samples. (C) Proteins upregulated and downregulated by hsa‐miR‐148a‐3p. (D) Proteins regulated by hsa‐miR‐142‐3p, hsa‐miR‐199b‐3p, hsa‐miR‐221‐3p, hsa‐miR‐222‐3p, and hsa‐miR‐584‐5p.

EV‐associated miRNAs and proteins that were present at different levels in PDAC cancer and para‐cancer tissue or serum from PDAC survivors and normal donors were examined. Combined transcriptomic and proteomics analyses were performed to identify more specific biomarkers. Five down‐regulated miRNAs (hsa‐miR‐199b‐3p, hsa‐miR‐221‐3p, hsa‐miR‐222‐3p, hsa‐miR‐142‐3p, and hsa‐miR‐584‐5p) and one up‐regulated miRNAs (hsa‐miR‐148a‐3p) were designated for verification (Figure 6A,B). Up‐regulated hsa‐miR‐148a‐3p was analyzed with proteomics to determine five up‐ and six down‐regulated proteins (Figure 6C). Five down‐regulated miRNAs were analyzed with proteomics to identify eight up‐ and nine down‐regulated proteins (Figure 6D). We selected CALR, a protein co‐regulated by hsa‐miR‐148a‐3p and hsa‐miR‐142‐3p, and performed immunohistochemistry (IHC) in clinical samples to verify its expression. The findings showed that tumor tissues had much lower levels of CALR expression than nearby tissues (Figure S4).

### 3.6 Deregulated miRNAs and Decreases in miR142‐3p and miR148a‐3p in PDAC Tumor Tissues

**FIGURE 7:**
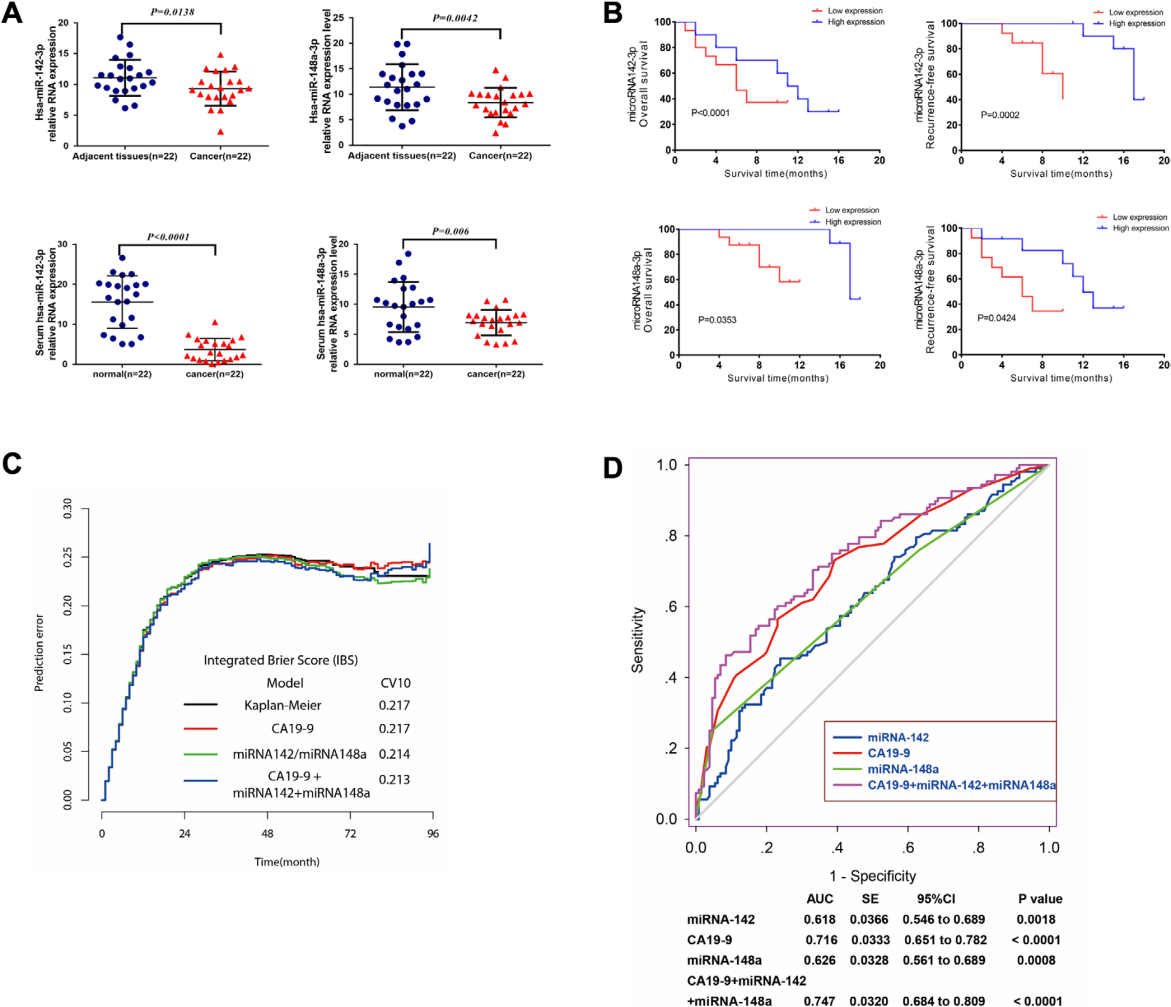
(A) MicroRNA 142‐3p and microRNA 148a‐3p expression consistently and significantly reduced in PDAC tumor tissues than in matched controls. (B) Kaplan–Meier survival curve generated with the median value of miR142‐3p and miR148a‐3p expression as the cut‐off. (C) Error curves for predictions made by different predictors. For the 18‐month follow‐up, the 10‐fold cross‐validated cumulative prediction error (PE) and apparent error (AE) were calculated. (D) AUC analysis based on the combination of miR142‐3p and miR148a‐3p‐based prediction and the CA199‐based model.

To analyze the miRNAs expression profile of pancreatic tissues, 3 pairs of pancreatic tissue samples (3 PDAC tissues and 3 matched non‐tumor pancreatic tissues) were assessed via transcriptomics and proteomics studies. When expression intensity sorting within PDAC non‐tumor and tumor groups was performed, when comparing PDAC tumor tissues to non‐tumor tissues, one miRNA was increased and five were decreased. Following this, the levels of the 6 miRNAs with the greatest number of alterations were verified. Prior to open pancreaticoduodenectomy (OPD), 10 patients were treated, while 12 underwent robotic pancreaticoduodenectomy (RPD). The characteristics of the baseline and demographic information are detailed in Table 3. A listing of the intraoperative factors and perioperative findings is depicted in Table 4. Through preliminary experiments, we identified the two miRNAs with the highest expression levels among the six tested miRNAs, specifically miR142‐3p and miR148a‐3p. We then collected clinical samples, performed fluorescence in situ hybridization (FISH), extracted EVs, and conducted RT‐qPCR to conduct a larger‐scale validation. The findings indicate that the levels of both miRNAs were considerably down‐regulated in cancerous tissues in contrast to surrounding tissues (Figure S5; Figure 7A). This result has also been verified in the blood of both healthy individuals and those diagnosed with pancreatic cancer, with the latter displaying a more noteworthy result.

**TABLE 4:** Intraoperative variables and perioperative outcomes.

Variate	26
Age, years, median (IQR)	64 (58 ~ 68)
Female, *n* (%)	10 (38.5%)
BMI, kg/m^2^, mean (SD)	22.5 (3.1)
Previous abdominal surgery, *n* (%)	3 (11.5%)
TB, μ mol/L, median (IQR)	16.4 (10.7 ~ 87.5)
Biliary drainage, n (%)	10 (38.5%)
CA199, U/mL, median (IQR)	231.9 (37.5 ~ 980.0)
Tumor size, cm, median (IQR)	3 (2.5 ~ 4)
PV/SMV resection, *n* (%)	3 (11.5%)
Year of diagnosis, *n* (%)	
2020	3 (11.5%)
2021	19 (73.1%)
2022	4 (15.4%)
Poor differentiation, *n* (%)	4 (15.4%)
Tumor stage, *n* (%)	
T1	3 (11.5%)
T2	15 (57.7%)
T3	4 (15.4%)
Lymph node stage, *n* (%)	
N0	9 (34.6%)
N1	9 (34.6%)
N2	5 (19.2%)
LVI, *n* (%)	13 (50.0%)
PNI, *n* (%)	19 (73.1%)
Adjuvant chemotherapy, *n* (%)	14 (53.8%)
Retrieved lymph nodes, median (IQR)	13 (7 ~ 21)
Positive lymph nodes, median (IQR)	1 (0 ~ 3)
LNR, median (IQR)	0 (0 ~ 0)
< 0.1	13 (50%)
≥ 0.1	13 (50%)
Ro resection, *n* (%)	26 (100%)
Blood loss, mL, median (IQR)	300 (100 ~ 500)
Operative time, min, median (IQR)	408 (344 ~ 477)
POD, *d*, median (IQR)	13 (10 ~ 20)
Reoperation, *n* (%)	2 (7.7%)
30‐day mortality, *n* (%)	0 (0%)
90‐day mortality, *n* (%)	0 (0%)
POPF	8 (30.8%)
Biochemical leak, *n* (%)	4 (15.4%)
CR‐POPF, *n* (%)	4 (15.4%)
Grade B	4 (15.4%)
Grade C	0 (0%)
Biliary fistula, *n* (%)	0 (0%)
Anastomotic fistula, *n* (%)	0 (0%)
PPH, *n* (%)	5 (19.2%)
Infection, *n* (%)	2 (7.7%)
DGE, *n* (%)	3 (11.5%)
Others, *n* (%)	1 (3.8%)
Total, *n* (%)	15 (57.7%)

### 3.7 A Poor Prognosis for PDAC Patients Is Highly Linked With Decreased miR142‐3p and miR148a‐3pexpression

After decreased miR142‐3p and miR148a‐3p expression in PDAC tissues and serum was confirmed, we next assessed whether their decrease was linked with the PDAC patients prognosis. It was determined that decreased miR142‐3p and miR148a‐3p expression was markedly associated with poor PDAC patients prognosis, as indicated by the Kaplan–Meier survival curve generated with the median values of miR142‐3p and miR148a‐3p levels as the optimal cut‐off (Figure 7B).

Moreover, the K‐adaptive partitioning statistical (kaps) algorithm implemented in R software was employed to find the prime cut‐off level for serum miR142‐3p and miR148a‐3p levels (7.9 and 2.9, respectively), which can significantly identify PDAC patients outcomes (Figure 7B). Altogether, these results suggest that reduced miR142‐3p and miR148a‐3p expression in PDAC is linked with poor prognosis, and miR142‐3p and miR148a‐3p expression values 7.9 and 2.9, respectively, can efficiently predict PDAC prognosis.

The accuracy of the ectopically expressed miRNA candidates was determined using prediction error curves to evaluate their pathologic and clinical value with the prognostic CA19‐9 level. The prediction error was computed over time with the help of the Brier score. Figure 7C illustrates that the combination of miR142‐3p and miR148a‐3p, and CA199 resulted in the lowest prediction error. Furthermore, as shown in Figure 7D, according to the ROC analysis, the AUC of the predictions based on miR142‐3p and miR148a‐3p in conjunction with the CA199 model (0.747) was higher than that of the CA199 model by itself (0.716). The outcomes of this study demonstrated that the predictive accuracy of clinical outcomes was higher when miR142‐3p and miR148a‐3p, and CA199 were combined, as opposed to CA199 alone.

## 4 Discussion

The analysis of EVs in cancer body fluid biopsy has obvious advantages of specificity, stability, and active molecular content. These can stably carry bioactive molecules such as miRNA, mRNA, DNA, and protein from tumor cells. There are more than 109 EVs in tumor cells per mL of blood, in contrast to the 1–10 circulating tumor cells (CTCs) per mL of blood. PDAC tissue‐derived EVs differ from serum EVs in terms of RNA type compositions, and Ti‐EVs can better reflect the pancreatic cancer tissue microenvironment. The expression pattern of pancreatic cancer‐related miRNAs in the tissues and serum of patients provides a reference for RNA‐related biological pathways during the disease process. Compared with healthy donors, there is a certain correlation between tissue EVs and circulating EVs, which provides a basis for the early detection of pancreatic cancer in peripheral blood.

Rather from being a simple reflection of the composition of their parent cells, a number of studies have suggested that exosomes are enriched in different repositories of miRNAs and that the selection and integration of miRNAs into them is not random [14, 15, 16, 17]. Moreover, exosomal miRNA profiles are triggers and cell type‐specific, representing the state of their parental cells [18, 19]. A previous study indicated that the specific profiles of miRNAs found in exosomes derived from tumor cells can differ due to the heterogeneity of the tumor and the complexity of the microenvironment, this might involve acid, hypoxia, and other stressors [20, 21]. The current results, however, showed that the exosomal miRNA level was varied in PDAC patients but homogeneous in healthy individuals. Although it is difficult to separate tumor‐derived exosomes from patient blood using existing techniques, liquid biopsy may be utilized for tumor detection and screening by utilizing the traits of exosomes that exhibit tumor heterogeneity and specificity.

Emerging research studies have established the efficacy of miRNAs as prognostic and diagnostic indicators for a wide range of diseases, with exosomal and circulating miRNAs serving as prime examples. Despite numerous studies supporting the use of isolated cellular miRNAs, recent findings have emphasized the possibility of miRNA panels, which comprise combinations of miRNAs, as diagnostic and prognostic biomarkers. The specificity of PDAC detection greatly improved with miRNA combinations, as opposed to individual miRNAs, consistent with our findings. KEGG and GO analyses additionally provided confirmation that our miRNA panel was linked to a variety of oncogene pathways. Considering the ductal origin and expression of neuroendocrine differentiation markers in PDAC, it is possible that our miRNA panel might have a role in the tumorigenesis and growth of PDAC. Our miRNA panel revealed new perspectives into the processes underlying the pathogenesis of PDAC. In brief, our research has yielded exosomal miRNAs that are specific to PDAC and have been analyzed as a potential prognostic indicator for this disease. A diagnostic and predictive indication for PDAC might belong to the 2‐miRNA panel, consisting of miR142‐3p and miR148a‐3p. Additional verification in a more extensive cohort and a greater understanding of the pathophysiological effects of these microRNAs are necessary to enhance the diagnostic precision of the 2‐miRNA panel.

Notably, miR‐142‐3p has a complex involvement in different kinds of tumors. In certain malignancies, it suppresses tumor growth by preventing invasion, metastasis, and proliferation of tumor cells [22, 23, 24]. However, contrasting evidence suggests that elevated miR‐142‐3p expression levels correlate with increased malignancy and poorer prognosis in other tumor types, with breast cancer demonstrating the most prominent expression pattern [25, 26]. Other reports have consistently reported a significant down‐regulation of miR‐142‐3p in association with the development, invasion, and metastasis of PDAC. Through the complex regulation of several target genes, including p53‐related genes, HSP70, and others, miR‐142‐3p has been demonstrated to have a significant influence on the invasion, proliferation, and metastatic potential of PDAC cells [3, 27]. Furthermore, the restoration of miR‐142‐3p expression has consistently demonstrated the suppression of PDAC cell propagation, penetration, and dissemination, along with the promotion of cell death. Concurrently, miR‐148a‐3p is consistently down‐regulated across a diverse array of cancer types and is closely associated with tumorigenesis, invasion, and metastasis. The diminished expression of miR‐148a‐3p has been observed to correspond with the increased expression of several target genes, including ITGA5, MAP3K9, and SLC7A11 [28, 29, 30]. Moreover, miR 148a‐3p suppresses the proliferation, invasion, and stemness characteristics of pancreatic cancer cells by inhibiting the Wnt1/β‐catenin pathway, which is controlled by Wnt1 [31]. The results obtained indicate that miR 148a‐3p might be a viable therapeutic target and predictive biomarker for pancreatic cancer.

In a previous study, it was reported that by combining different miRNAs as biomarkers to reveal their unique diagnostic performance for pancreatic cancer, they evaluated the AUC of 0.93 for the diagnosis of PDAC with a combination of 3 miRNAs, which was significantly higher compared to the AUC values of 0.64, 0.76, and 0.74 for the diagnosis of PDAC with a single miRNA [32]. Similarly, in this study, the AUC value for PDAC diagnosis using CA‐199 alone was only 0.716, whereas the AUC value for PDAC diagnosis using a combination of CA‐199, miR142‐3p, and miR148a‐3p was 0.747, which significantly improved the diagnostic performance. It is worth noting that a simple blood test that is not only robust but also non‐invasive can greatly help detect PDAC early. A serum protein signature that could predict clinical PDAC outcome compared to CA199 alone was identified. Using this signature together with routine clinical parameters, a reliable model that predicted prognosis‐stratified PDAC was developed and validated. This model has more potential than the current standard CA199, with 0.86 accuracy. Another study on PDAC fluid determined biomarkers related to prognosis with comparable prediction accuracies to our serum‐based strategy. The detection of our distinctive serum miRNA signature only requires a small blood volume, which is easily accessible preoperatively.

Given the clinical, RNA‐ sequencing, transcriptomic, and proteomics analyses, biochemical, and functional significance of exosomal miR142‐3p and miR148a‐3p in PDAC, miR142‐3p, and miR148a‐3p were found to be essential for pancreatic carcinogenesis, and addressing these genes may be essential for PDAC prevention or therapy. Further investigations with larger cohorts are needed for verification and improving the diagnostic accuracy of the 2‐miRNA panel.

## 5 Limitations

The study's retrospective methodology, limited sample size, and inclusion of pancreatic resection PDAC patients from a single institution are among its drawbacks, despite the encouraging results. Selection bias may result because of the inclusion criteria of selecting participants who had pathologically confirmed PDAC and were undergoing surgical resection. More validation investigations on a larger study size are needed for further evaluation of the diagnostic accuracy of this EVs classifier and multiparameter model. Current data suggest that the application of molecular diagnostics in clinical settings could result in a higher certainty in clinical prognosis for PDAC.

## 6 Recommendations

As a mature secretory signal transduction vector, EVs have been isolated and purified. As a nano biofilm structure, EVs can better ensure the integrity and biological activity of the contents and greatly improve their feasibility as a diagnostic marker for PDAC. As more research is carried out, secreted miRNAs may become new diagnostic markers for pancreatic cancer and novel therapeutic targets. In the future, follow‐up investigations can be performed from the following two aspects: (1) explore mature and reliable monitoring technology to further clarify the types and biological characteristics of miRNAs related to pancreatic cancer in EVs. (2) Take tissue‐specific miRNAs in EVs and construct the target gene target miRNAs protein network, identify the pathogenesis of pancreatic cancer at gene and protein levels, to further improve diagnosis and treatment of pancreatic cancer.

EV is a novel biomarker, non‐invasive liquid biopsy able to predict disease progression or response to therapy.

## Author Contributions

**Qian Zhu:** conceptualization (lead), data curation (lead), software (lead), writing – original draft (lead). **Zhang Chen:** resources (supporting), software (supporting). **Ming Tian:** data curation (supporting), methodology (supporting), resources (supporting), validation (supporting), writing – review and editing (equal). **Xin Yan:** resources (supporting), software (supporting). **Xiangdong Gongye:** data curation (supporting). **Zhicheng Liu:** data curation (supporting). **Anbang Zhao:** data curation (supporting), methodology (supporting), resources (supporting). **Zhiyong Yang:** conceptualization (equal), supervision (lead), writing – review and editing (lead). **Yufeng Yuan:** conceptualization (equal), supervision (lead), writing – review and editing (lead).

## Ethics Statement

The study was approved by the Ethics Committee of the Zhongnan Hospital of Wuhan University, and the study enrollment was carried out after a written informed consent was obtained from each patient. Our research has been carried out in accordance with The Code of Ethics of the World Medical Association (Declaration of Helsinki).

## Consent

We confirm that this manuscript has not been published elsewhere and is not consideration by another journal. All authors have approved the manuscript and agree with submission to Clinical Cancer Research. And he study enrollment was carried out after a written informed consent was obtained from each patient.

## Conflicts of Interest

The authors declare no conflicts of interest.

## Supporting information


Data S1.

## Data Availability

The data of this study are included in this article and its online Supporting Information Files. Please contact the corresponding author if any further information is required.
