# Supplementary material for: Improved Predictability of Diagnosis and Prognosis Using Serum‐ and Tissue‐Derived Extracellular Vesicles From Bulk mRNA Sequencing in Pancreatic Ductal Adenocarcinoma
Source: Cancer Med. 2025 Jan 15;14(2):e70538. doi: 10.1002/cam4.70538 (PMC11733676; doi:10.1002/cam4.70538)
Supplement: Supplementary file 1 — Data S1. [file CAM4-14-e70538-s001.docx]

**Improved predictability of diagnosis and prognosis using serum and tissue derived extracellular vesicles from bulk mRNA sequencing in pancreatic ductal adenocarcinoma**

Qian Zhu1,2†, Zhang Chen1†, Ming Tian 1,2†, Xin Yan1, Xiangdong Gongye1, Zhicheng Liu1, Anbang Zhao1, Zhiyong Yang1,2*, Yufeng Yuan 1,2,3*

1 Department of Hepatobiliary and Pancreatic Surgery, Zhongnan Hospital of Wuhan University, Wuhan, Hubei, 430071, People's Republic of China.

2 Clinical Medicine Research Center for Minimally Invasive Procedure of Hepatobiliary & Pancreatic Diseases of Hubei Province, Wuhan, Hubei, 430071, People's Republic of China.

3 TaiKang Center for Life and Medical Sciences, Wuhan University, Wuhan, 430071, People's Republic of China.† These authors have contributed equally to this work.

***Correspondence :**

Yufeng Yuan, yuanyf1971@whu.edu.cn

Zhiyong Yang, yangzhiyong@whu.edu.cn

**SUPPLEMENTARY MATERIALS AND METHODS**

**IHC**

Briefly, both surrounding and tumor tissues were fixed with 4% paraformaldehyde, blocked with 10% FBS, and permeabilized with Triton X-100. Sections were incubated with CALR antibody (Proteintech, 1:50) overnight at 4°C. After washing with PBS, sections were incubated with secondary antibodies for 1 hour at room temperature. After counterstaining with hematoxylin, sections were observed under a microscope.

**FISH**

Paraffin sections were dewaxed into water and then digested. After pre-hybridization of the sections, hybridization solution containing 1μM probe was added dropwise and hybridized overnight in a 37°C incubator (hsa-miR-142-3p and hsa-miR-148a-3p specific probes were synthesized by Siwega). Then we washed away the hybridization solution on the sections, rinsed them with PBS, added anti-fluorescence quenching mounting medium.Finally, observed and collected images under the fluorescence microscope.

Table S1. Unannotated reads of biotype RNA distribution from each sample.

| Sample ID | Clean_reads | rRNA | snRNA | snoRNA | tRNA | Repbase | miRNA | Others |
| --- | --- | --- | --- | --- | --- | --- | --- | --- |
| Healthy-serum-1 | 6975639 | 3601108 | 1 | 1115 | 287474 | 92975 | 368786 | 2624180 |
| Healthy-serum-2 | 6184756 | 2944448 | 10 | 1195 | 231934 | 127622 | 734816 | 2144731 |
| Healthy-serum-3 | 8032169 | 2616043 | 7 | 1305 | 212657 | 247985 | 1870383 | 3083789 |
| N1 | 8120525 | 4257605 | 13 | 19060 | 483530 | 224747 | 389986 | 2745584 |
| N2 | 10543597 | 6976315 | 2 | 20102 | 1089025 | 118346 | 633267 | 1706540 |
| N3 | 8493302 | 4707637 | 0 | 15130 | 949015 | 160937 | 336306 | 2324277 |
| N4 | 5386474 | 2668388 | 10 | 7558 | 271514 | 75163 | 553076 | 1810765 |
| N5 | 17398140 | 9414585 | 14 | 19195 | 1170548 | 238822 | 2766492 | 3788484 |
| N6 | 7398891 | 3468767 | 23 | 9622 | 644213 | 103680 | 846665 | 2325921 |
| N7 | 17090183 | 4001046 | 17 | 69010 | 761332 | 260205 | 8084713 | 3913860 |
| N8 | 7882494 | 4093709 | 3 | 19332 | 428724 | 86600 | 1637387 | 1616739 |
| PC-serum-1 | 6538663 | 3003276 | 0 | 968 | 206268 | 69154 | 837574 | 2421423 |
| PC-serum-2 | 6013762 | 2535698 | 0 | 1632 | 196774 | 55431 | 1044626 | 2179601 |
| PC-serum-3 | 4042804 | 2012188 | 0 | 1205 | 142312 | 41651 | 404965 | 1440483 |
| T1 | 18064139 | 6996502 | 13 | 50240 | 992290 | 323971 | 5027985 | 4673138 |
| T2 | 9976403 | 3808199 | 21 | 27628 | 1448651 | 222607 | 1806712 | 2662585 |
| T3 | 9852103 | 4583531 | 3 | 18961 | 1124505 | 184314 | 1263657 | 2677132 |
| T4 | 6914595 | 2676855 | 4 | 12781 | 1387067 | 124688 | 644494 | 2068706 |
| T5 | 13316956 | 5792380 | 9 | 20946 | 1031075 | 132372 | 3075550 | 3264624 |
| T6 | 7619244 | 3669867 | 41 | 6316 | 684825 | 79759 | 395136 | 2783300 |
| T7 | 18025131 | 4519157 | 28 | 36676 | 582590 | 197445 | 7176660 | 5512575 |
| T8 | 8828638 | 3792522 | 0 | 16453 | 674800 | 85261 | 1920103 | 2339499 |


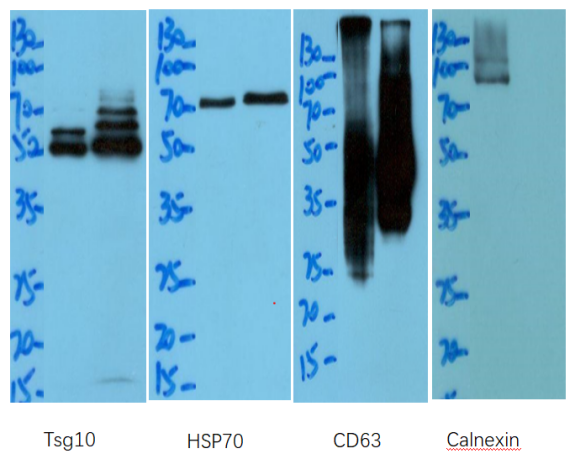

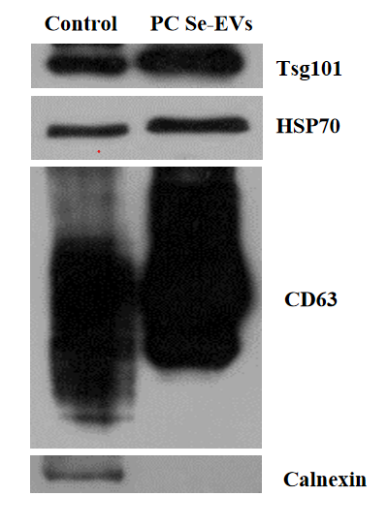


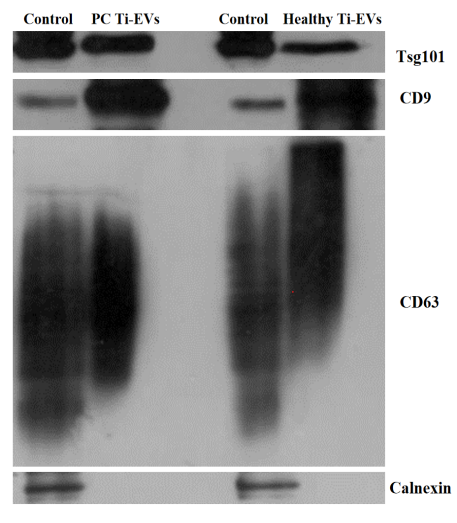

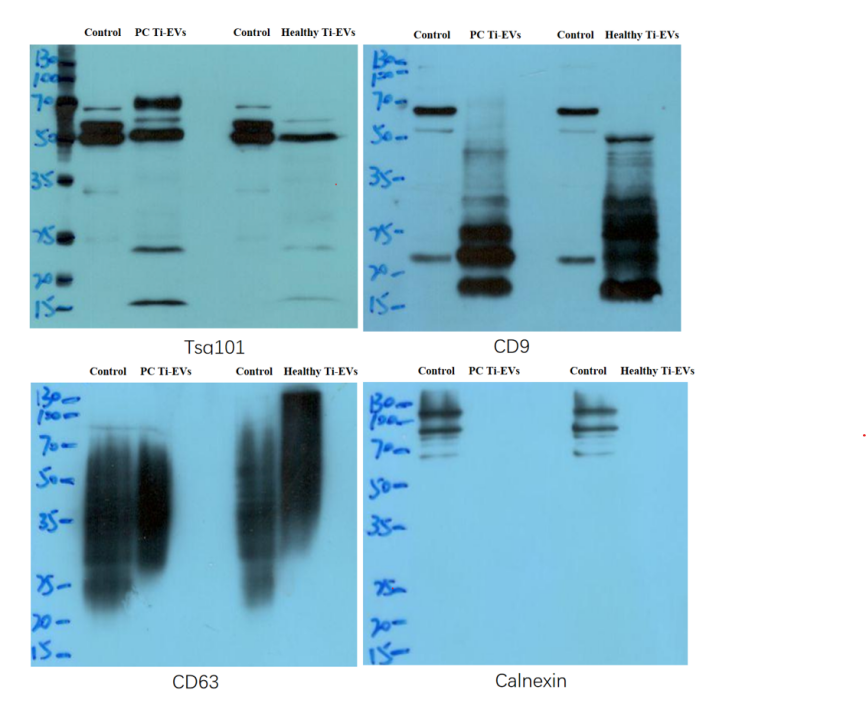


Figure S1.The full length original blots of WB analyses of Ti-EVs and Se-EVs in different participates.TSG101, HSP70, and CD63 were highly expressed in Se-EVs, while Ti-EVs were enriched with TSG101, CD9, and CD63


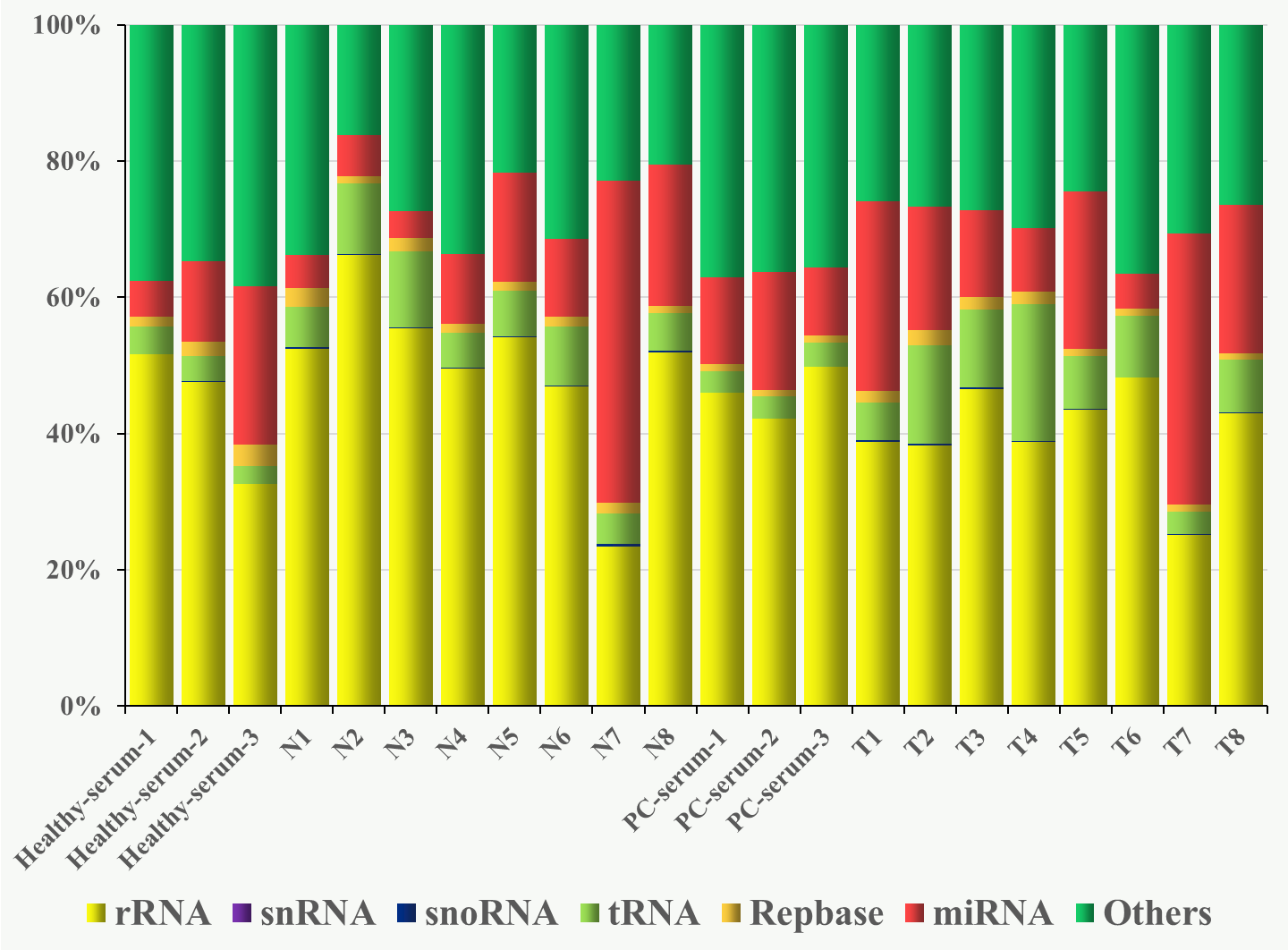


Figure S2. Percentage of biotype RNA counts in each sample. This graph represents average percentage of biotype counts of each sample from Ti-EVs and Se-EVs.


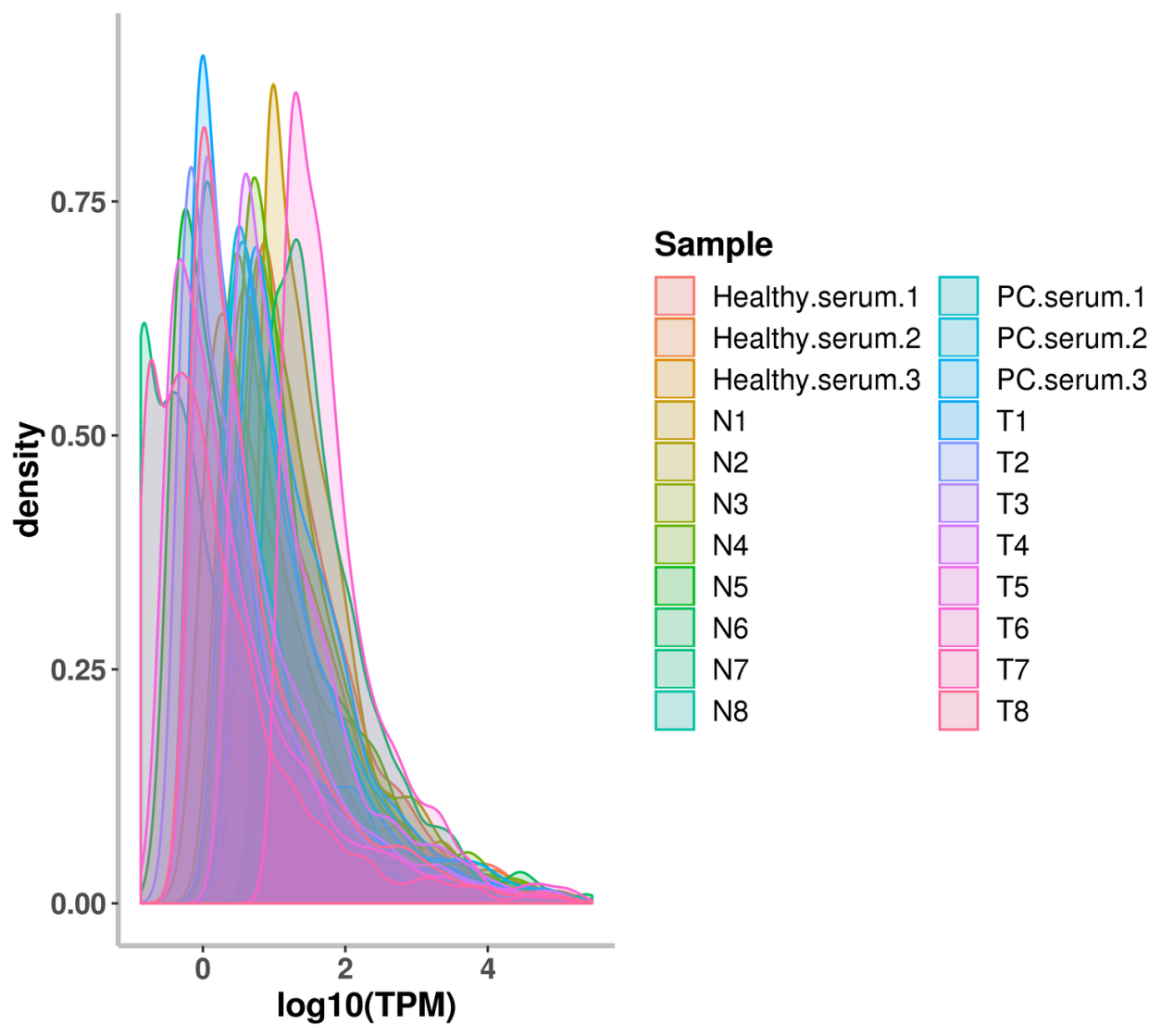


Figure S3. The overall distribution map of miRNA expression


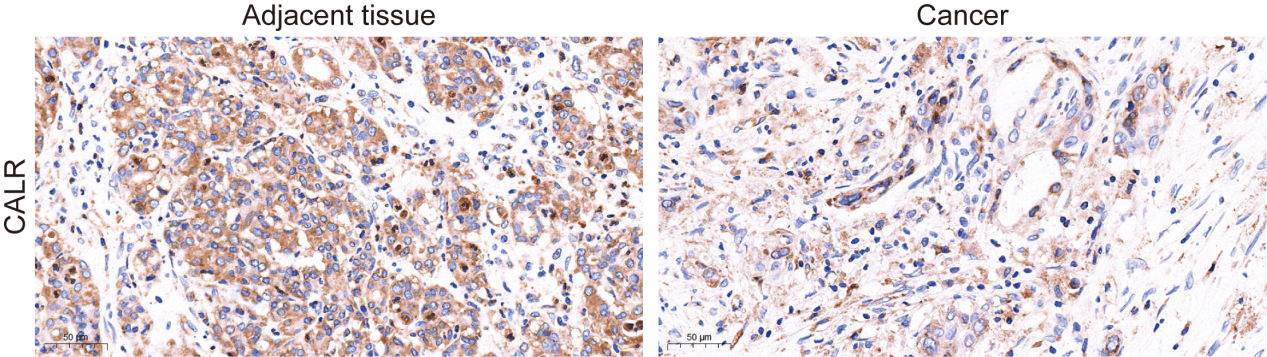


Figure S4. IHC showed the expression of CALR in tumor tissue and surrounding tissues. (Scale bar = 50 μm)


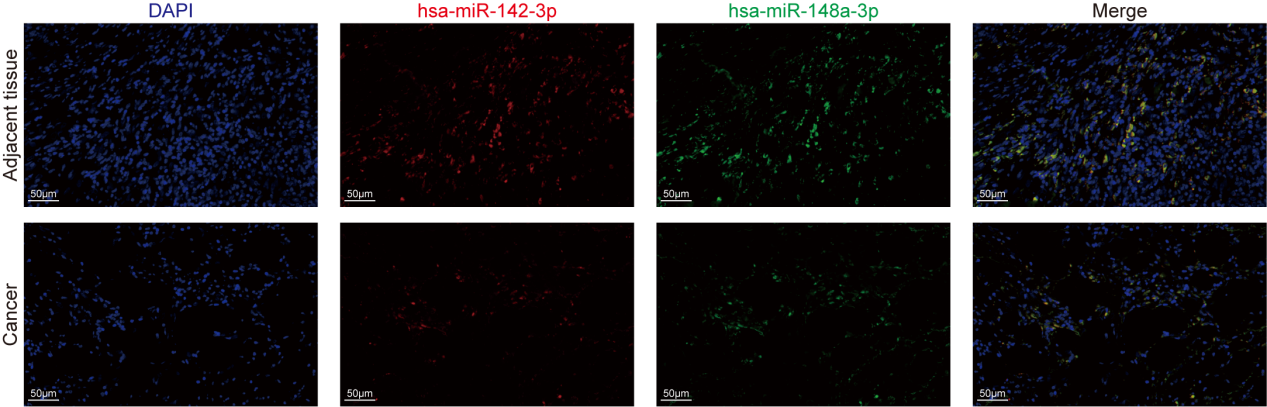


Figure S5. FISH showed the expression of hsa-miR-142-3p and hsa-miR-148a-3p in tumor tissue and surrounding tissues. Hsa-miR-142-3p was labeled with Cy3 (red), hsa-miR-148a-3p was labeled with FITC (green) and nuclei were stained with DAPI (blue). (Scale bar = 50 μm)

| **Type** | **COG** | **GO** | **KEGG** | **KOG** | **NR** | **Pfam** | **Swiss-Prot** | **eggNOG** |
| --- | --- | --- | --- | --- | --- | --- | --- | --- |
| G1_vs_G2 | 4979 | 14831 | 15293 | 10800 | 15785 | 14839 | 15734 | 15448 |
| G2_vs_G4 | 5918 | 17525 | 18118 | 12618 | 18751 | 17554 | 18661 | 18317 |
| G3_vs_G4 | 3727 | 11181 | 11555 | 8130 | 11922 | 11159 | 11872 | 11684 |

Table S2. Statistics of the number of annotated differential miRNA target genes.

Classifiers constructed from six identified DEMs.

| **Classifier** | **Elements** |
| --- | --- |
| 1 | \| hsa.miR.584.5p \| \| --- \| \| hsa.miR.142.3p \| \| hsa.miR.148a.3p \| \| hsa.miR.222.3p \| \| hsa.miR.221.3p \| \| hsa.miR.199b.3p \| |
| 2 | \| hsa.miR.584.5p+hsa.miR.142.3p \| \| --- \| \| hsa.miR.584.5p+hsa.miR.148a.3p \| \| hsa.miR.584.5p+hsa.miR.222.3p \| \| hsa.miR.584.5p+hsa.miR.221.3p \| \| hsa.miR.584.5p+hsa.miR.199b.3p \| \| hsa.miR.142.3p+hsa.miR.148a.3p \| \| hsa.miR.142.3p+hsa.miR.222.3p \| \| hsa.miR.142.3p+hsa.miR.221.3p \| \| hsa.miR.142.3p+hsa.miR.199b.3p \| \| hsa.miR.148a.3p+hsa.miR.222.3p \| \| hsa.miR.148a.3p+hsa.miR.221.3p \| \| hsa.miR.148a.3p+hsa.miR.199b.3p \| \| hsa.miR.222.3p+hsa.miR.221.3p \| \| hsa.miR.222.3p+hsa.miR.199b.3p \| \| hsa.miR.221.3p+hsa.miR.199b.3p \| |
| 3 | \| hsa.miR.584.5p+hsa.miR.142.3p+hsa.miR.148a.3p \| \| --- \| \| hsa.miR.584.5p+hsa.miR.142.3p+hsa.miR.222.3p \| \| hsa.miR.584.5p+hsa.miR.142.3p+hsa.miR.221.3p \| \| hsa.miR.584.5p+hsa.miR.142.3p+hsa.miR.199b.3p \| \| hsa.miR.584.5p+hsa.miR.148a.3p+hsa.miR.222.3p \| \| hsa.miR.584.5p+hsa.miR.148a.3p+hsa.miR.221.3p \| \| hsa.miR.584.5p+hsa.miR.148a.3p+hsa.miR.199b.3p \| \| hsa.miR.584.5p+hsa.miR.222.3p+hsa.miR.221.3p \| \| hsa.miR.584.5p+hsa.miR.222.3p+hsa.miR.199b.3p \| \| hsa.miR.584.5p+hsa.miR.221.3p+hsa.miR.199b.3p \| \| hsa.miR.142.3p+hsa.miR.148a.3p+hsa.miR.222.3p \| \| hsa.miR.142.3p+hsa.miR.148a.3p+hsa.miR.221.3p \| \| hsa.miR.142.3p+hsa.miR.148a.3p+hsa.miR.199b.3p \| \| hsa.miR.142.3p+hsa.miR.222.3p+hsa.miR.221.3p \| \| hsa.miR.142.3p+hsa.miR.222.3p+hsa.miR.199b.3p \| \| hsa.miR.142.3p+hsa.miR.221.3p+hsa.miR.199b.3p \| \| hsa.miR.148a.3p+hsa.miR.222.3p+hsa.miR.221.3p \| \| hsa.miR.148a.3p+hsa.miR.222.3p+hsa.miR.199b.3p \| \| hsa.miR.148a.3p+hsa.miR.221.3p+hsa.miR.199b.3p \| \| hsa.miR.222.3p+hsa.miR.221.3p+hsa.miR.199b.3p \| |
| 4 | \| hsa.miR.584.5p+hsa.miR.142.3p+hsa.miR.148a.3p+hsa.miR.222.3p \| \| --- \| \| hsa.miR.584.5p+hsa.miR.142.3p+hsa.miR.148a.3p+hsa.miR.221.3p \| \| hsa.miR.584.5p+hsa.miR.142.3p+hsa.miR.148a.3p+hsa.miR.199b.3p \| \| hsa.miR.584.5p+hsa.miR.142.3p+hsa.miR.222.3p+hsa.miR.221.3p \| \| hsa.miR.584.5p+hsa.miR.142.3p+hsa.miR.222.3p+hsa.miR.199b.3p \| \| hsa.miR.584.5p+hsa.miR.142.3p+hsa.miR.221.3p+hsa.miR.199b.3p \| \| hsa.miR.584.5p+hsa.miR.148a.3p+hsa.miR.222.3p+hsa.miR.221.3p \| \| hsa.miR.584.5p+hsa.miR.148a.3p+hsa.miR.222.3p+hsa.miR.199b.3p \| \| hsa.miR.584.5p+hsa.miR.148a.3p+hsa.miR.221.3p+hsa.miR.199b.3p \| \| hsa.miR.584.5p+hsa.miR.222.3p+hsa.miR.221.3p+hsa.miR.199b.3p \| \| hsa.miR.142.3p+hsa.miR.148a.3p+hsa.miR.222.3p+hsa.miR.221.3p \| \| hsa.miR.142.3p+hsa.miR.148a.3p+hsa.miR.222.3p+hsa.miR.199b.3p \| \| hsa.miR.142.3p+hsa.miR.148a.3p+hsa.miR.221.3p+hsa.miR.199b.3p \| \| hsa.miR.142.3p+hsa.miR.222.3p+hsa.miR.221.3p+hsa.miR.199b.3p \| \| hsa.miR.148a.3p+hsa.miR.222.3p+hsa.miR.221.3p+hsa.miR.199b.3p \| |
| 5 | \| hsa.miR.584.5p+hsa.miR.142.3p+hsa.miR.148a.3p+hsa.miR.222.3p+hsa.miR.221.3p \| \| --- \| \| hsa.miR.584.5p+hsa.miR.142.3p+hsa.miR.148a.3p+hsa.miR.222.3p+hsa.miR.199b.3p \| \| hsa.miR.584.5p+hsa.miR.142.3p+hsa.miR.148a.3p+hsa.miR.221.3p+hsa.miR.199b.3p \| \| hsa.miR.584.5p+hsa.miR.142.3p+hsa.miR.222.3p+hsa.miR.221.3p+hsa.miR.199b.3p \| \| hsa.miR.584.5p+hsa.miR.148a.3p+hsa.miR.222.3p+hsa.miR.221.3p+hsa.miR.199b.3p \| \| hsa.miR.142.3p+hsa.miR.148a.3p+hsa.miR.222.3p+hsa.miR.221.3p+hsa.miR.199b.3p \| |
| 6 | hsa.miR.584.5p+hsa.miR.142.3p+hsa.miR.148a.3p+hsa.miR.222.3p+hsa.miR.221.3p+hsa.miR.199b.3p |

Target genes of the six EV-miRNAs

| miRNA | targetgene | protein |
| --- | --- | --- |
| hsa-miR-148a-3p | CANX | P27824 |
| hsa-miR-222-3p | GNAI2 | P04899 |
| hsa-miR-148a-3p | LTBP1 | Q14766 |
| hsa-miR-221-3p | GNAI2 | P04899 |
| hsa-miR-148a-3p | HSP90B1 | P14625 |
| hsa-miR-222-3p | THBS1 | P07996 |
| hsa-miR-148a-3p | CGGBP1 | Q9UFW8 |
| hsa-miR-221-3p | ANXA3 | P12429 |
| hsa-miR-221-3p | PELI1 | Q96FA3 |
| hsa-miR-221-3p | THBS1 | P07996 |
| hsa-miR-222-3p | PELI1 | Q96FA3 |
| hsa-miR-142-3p | PELI1 | Q96FA3 |
| hsa-miR-199b-3p | LTBP1 | Q14766 |
| hsa-miR-148a-3p | CALR | P27797 |
| hsa-miR-148a-3p | GNB1 | P62873 |
| hsa-miR-148a-3p | MSN | P26038 |
| hsa-miR-221-3p | GNB1 | P62873 |
| hsa-miR-221-3p | HEG1 | Q9ULI3 |
| hsa-miR-221-3p | MSN | P26038 |
| hsa-miR-222-3p | CANX | P27824 |
| hsa-miR-222-3p | HEG1 | Q9ULI3 |
| hsa-miR-142-3p | C4BPB | P20851 |
| hsa-miR-584-5p | HSPA5 | P11021 |
| hsa-miR-199b-3p | HLA-B | P01889 |
| hsa-miR-199b-3p | THBS1 | P07996 |
| hsa-miR-148a-3p | GNAI2 | P04899 |
| hsa-miR-148a-3p | HLA-B | P01889 |
| hsa-miR-148a-3p | HPX | P02790 |
| hsa-miR-148a-3p | THBS1 | P07996 |
| hsa-miR-148a-3p | TUBB | P07437 |
| hsa-miR-221-3p | CANX | P27824 |
| hsa-miR-221-3p | HLA-B | P01889 |
| hsa-miR-221-3p | HSP90B1 | P14625 |
| hsa-miR-221-3p | LGALS3BP | Q08380 |
| hsa-miR-222-3p | CANX | P27824 |
| hsa-miR-222-3p | HSPA5 | P11021 |
| hsa-miR-222-3p | LGALS3BP | Q08380 |
| hsa-miR-222-3p | RPL12 | P30050 |
| hsa-miR-142-3p | CALR | P27797 |
| hsa-miR-584-5p | LUM | P51884 |
| hsa-miR-199b-3p | HEG1 | Q9ULI3 |
| hsa-miR-199b-3p | HLA-B | P01889 |
| hsa-miR-199b-3p | HLA-B | P01889 |
| hsa-miR-199b-3p | HLA-B | P01889 |
| hsa-miR-199b-3p | HLA-B | P01889 |
| hsa-miR-199b-3p | HLA-B | P01889 |
| hsa-miR-199b-3p | MSN | P26038 |


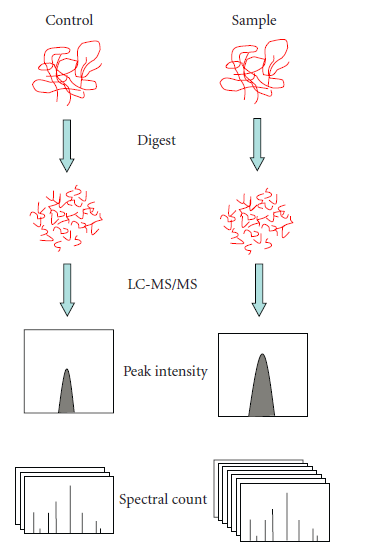


Label free methods.

Expression of miRNA142-3p and miRNA 148a-3p in RT-qPCR in 4 groups of samples.

| Serum hsa-miR-148-3p | | Serum hsa-miR-142-3p | | Tissues hsa-miR-148-3p | | Tissues hsa-miR-142-3p | | Serum hsa-miR-148-3p | | Serum hsa-miR-142-3p | | Tissues hsa-miR-148-3p | | Tissues hsa-miR-142-3p | |
| --- | --- | --- | --- | --- | --- | --- | --- | --- | --- | --- | --- | --- | --- | --- | --- |
| normal(n=22) | cancer(n=22) | normal(n=22) | cancer(n=22) | Adjacent tissues(n=19) | Cancer(n=19) | Adjacent tissues(n=19) | Cancer(n=19) | normal(n=22) | cancer(n=22) | normal(n=22) | cancer(n=22) | Adjacent tissues(n=19) | Cancer(n=19) | Adjacent tissues(n=19) | Cancer(n=19) |
| 3.7 | 3.79 | 16.63 | 1.27 | 8.563 | 2.441 | 6.56 | 5.84 | 3.7 | 3.79 | 16.63 | 1.27 | 8.563 | 2.441 | 6.56 | 5.84 |
| 4.2 | 3.65 | 6.76 | 5.18 | 5.169 | 4.13 | 11.97 | 7.27 | 4.2 | 3.65 | 6.76 | 5.18 | 5.169 | 4.13 | 11.97 | 7.27 |
| 10.73 | 4.78 | 17.5 | 7.41 | 11.007 | 13.271 | 10.33 | 12.57 | 10.73 | 4.78 | 17.5 | 7.41 | 11.007 | 13.271 | 10.33 | 12.57 |
| 10.21 | 8.16 | 9.78 | 6 | 14.045 | 7.897 | 17.67 | 5.87 | 10.21 | 8.16 | 9.78 | 6 | 14.045 | 7.897 | 17.67 | 5.87 |
| 8 | 7.6 | 20.09 | 0.77 | 9.049 | 9.916 | 9.37 | 9.46 | 8 | 7.6 | 20.09 | 0.77 | 9.049 | 9.916 | 9.37 | 9.46 |
| 10.73 | 6.73 | 20.26 | 0.48 | 13.973 | 7.069 | 10.99 | 8.43 | 10.73 | 6.73 | 20.26 | 0.48 | 13.973 | 7.069 | 10.99 | 8.43 |
| 4.54 | 7.17 | 5.08 | 2.12 | 12.768 | 9.983 | 9.46 | 10.46 | 4.54 | 7.17 | 5.08 | 2.12 | 12.768 | 9.983 | 9.46 | 10.46 |
| 12.77 | 5.72 | 22.57 | 5.19 | 19.884 | 4.472 | 16.48 | 14.84 | 12.77 | 5.72 | 22.57 | 5.19 | 19.884 | 4.472 | 16.48 | 14.84 |
| 12.6 | 3.45 | 15.59 | 4.75 | 10.68 | 10.176 | 14.3 | 10.45 | 12.6 | 3.45 | 15.59 | 4.75 | 10.68 | 10.176 | 14.3 | 10.45 |
| 14.39 | 10.54 | 5.08 | 2.32 | 9.162 | 10.123 | 8.94 | 8.24 | 14.39 | 10.54 | 5.08 | 2.32 | 9.162 | 10.123 | 8.94 | 8.24 |
| 13.96 | 6.75 | 15.7 | 0.95 | 4.74 | 7.325 | 7.46 | 2.39 | 13.96 | 6.75 | 15.7 | 0.95 | 4.74 | 7.325 | 7.46 | 2.39 |
| 10.05 | 6.37 | 19.94 | 1.52 | 11.932 | 6.434 | 6.18 | 9.79 | 10.05 | 6.37 | 19.94 | 1.52 | 11.932 | 6.434 | 6.18 | 9.79 |
| 9.56 | 8.32 | 11.63 | 5.91 | 13.213 | 9.614 | 12.65 | 9.38 | 9.56 | 8.32 | 11.63 | 5.91 | 13.213 | 9.614 | 12.65 | 9.38 |
| 9.74 | 9.5 | 19.55 | 4.58 | 11.868 | 11.347 | 12.92 | 12.35 | 9.74 | 9.5 | 19.55 | 4.58 | 11.868 | 11.347 | 12.92 | 12.35 |
| 10.43 | 10.74 | 11.23 | 2.94 | 17.901 | 9.94 | 9.75 | 9.09 | 10.43 | 10.74 | 11.23 | 2.94 | 17.901 | 9.94 | 9.75 | 9.09 |
| 16.94 | 7.71 | 22.33 | 10.59 | 15.731 | 9.798 | 11.45 | 12.19 | 16.94 | 7.71 | 22.33 | 10.59 | 15.731 | 9.798 | 11.45 | 12.19 |
| 18.41 | 7.83 | 23.09 | 0.09 | 13.947 | 8.422 | 11.53 | 9.11 | 18.41 | 7.83 | 23.09 | 0.09 | 13.947 | 8.422 | 11.53 | 9.11 |
| 6.21 | 8.18 | 7.32 | 6.29 | 3.755 | 8.114 | 10.89 | 10.49 | 6.21 | 8.18 | 7.32 | 6.29 | 3.755 | 8.114 | 10.89 | 10.49 |
| 6.62 | 8.11 | 26.64 | 6.82 | 19.833 | 14.799 | 14.79 | 12.87 | 6.62 | 8.11 | 26.64 | 6.82 | 19.833 | 14.799 | 14.79 | 12.87 |
| 5.87 | 7.38 | 19.1 | 2.72 |  |  |  |  | 5.87 | 7.38 | 19.1 | 2.72 |  |  |  |  |
| 3.68 | 3.3 | 6.46 | 1.77 |  |  |  |  | 3.68 | 3.3 | 6.46 | 1.77 |  |  |  |  |
| 6.53 | 6.93 | 19.8 | 1.08 |  |  |  |  | 6.53 | 6.93 | 19.8 | 1.08 |  |  |  |  |
